# In silico prediction of the enzymes involved in the degradation of the herbicide molinate by *Gulosibacter molinativorax* ON4^T^

**DOI:** 10.1038/s41598-022-18732-5

**Published:** 2022-09-15

**Authors:** A. R. Lopes, E. Bunin, A. T. Viana, H. Froufe, A. Muñoz-Merida, D. Pinho, J. Figueiredo, C. Barroso, I. Vaz-Moreira, X. Bellanger, C. Egas, O. C. Nunes

**Affiliations:** 1 LEPABE - Laboratory for Process Engineering, Environment, Biotechnology and Energy, Departamento de Engenharia Química, Faculty of Engineering, University of Porto, Rua Dr. Roberto Frias, 4200-465 Porto, Portugal; 2 Next Generation Sequencing Unit, Biocant, BiocantPark, Núcleo 04, Lote 8, 3060-197 Cantanhede, Portugal; 3 CIBIO – Research Centre in Biodiversity and Genetic Resources, InBIO, University of Porto, Rua Padre Armando Quintas, no. 7, 4485-661 Vairão, Portugal; 4 Center for Neuroscience and Cell Biology, University of Coimbra, 3004-504 Coimbra, Portugal; 5 Universidade Católica Portuguesa, CBQF – Centro de Biotecnologia e Química Fina – Laboratório Associado, Escola Superior de Biotecnologia, Rua Diogo Botelho 1327, 4169-005 Porto, Portugal; 6 Université de Lorraine, CNRS, LCPME, 54000 Nancy, France

**Keywords:** Microbiology, Environmental microbiology, Transcriptomics, Genomics, Mobile elements

## Abstract

*Gulosibacter molinativorax* ON4^T^ is the only known organism to produce molinate hydrolase (MolA), which catalyses the breakdown of the thiocarbamate herbicide into azepane-1-carboxylic acid (ACA) and ethanethiol. A combined genomic and transcriptomic strategy was used to fully characterize the strain ON4^T^ genome, particularly the *mol*A genetic environment, to identify the potential genes encoding ACA degradation enzymes. Genomic data revealed that *mol*A is the only catabolic gene of a novel composite transposon (Tn*6311*), located in a novel low copy number plasmid (pARLON1) harbouring a putative T4SS of the class FATA. pARLON1 had an ANI value of 88.2% with contig 18 from *Agrococcus casei* LMG 22410^T^ draft genome. Such results suggest that pARLON1 is related to genomic elements of other *Actinobacteria*, although Tn*6311* was observed only in strain ON4^T^. Furthermore, genomic and transcriptomic data demonstrated that the genes involved in ACA degradation are chromosomal. Based on their overexpression when growing in the presence of molinate, the enzymes potentially involved in the heterocyclic ring breakdown were predicted. Among these, the activity of a protein related to caprolactone hydrolase was demonstrated using heterologous expression. However, further studies are needed to confirm the role of the other putative enzymes.

## Introduction

Biocatalysis is a crucial component of white or industrial biotechnology, relying on the broad catalytic activity of enzymes leading to the production of value-added compounds (e.g., detergents, cosmetics), or catalyzing the degradation of several environmental contaminants (e.g., pesticides, hydrocarbons)^1–3^. Genome and transcriptome sequencing projects are critical to the burst of the biocatalysis field, enabling the identification of genes encoding proteins putatively involved in the production/degradation of the target compound(s), which can be further characterized to develop a biocatalytic solution^1,4,5^.

The biotechnological potential of *Actinobacteria* is known^1,3^, yet there is still much to unveil^6^. *Gulosibacter molinativorax* ON4^T^, an actinobacterium of the family *Microbacteriaceae*^7^, is an example. It was isolated from a five-membered enrichment culture (strains ON1-ON5) able to mineralize the thiocarbamate herbicide molinate^8,9^. Strain ON4^T^ produces molinate hydrolase (MolA), responsible for breaking molinate into azepane-1-carboxylic acid (ACA) and ethanethiol^8,10^. Strain ON4^T^ does not further use ethanethiol, but strains ON1 and ON2 consume this metabolite. In contrast, ACA supports the growth of strain ON4^T^ and other culture members^8^. Hence, molinate mineralization occurs through the cooperation between strain ON4^T^ and the culture members able to deplete the sulfur metabolite^8^. Although the putative degradation pathway for molinate has been described^8^, MolA is the only enzyme involved in this pathway identified and characterized so far^10–12^. Previous studies have shown the capacity of MolA and the recombinant mutant (Arg187Ala) thereof to degrade the thiocarbamate herbicides molinate and thiobencarb^10–12^. The biocatalytic potential of the enzymes involved in the ACA degradation pathway in *G. molinativorax* ON4^T^ motivated this study.

Genes encoding enzymes involved in a particular metabolic pathway, as in xenobiotic degradation, tend to be close to each other^13–16^, often organized in catabolic operons with common regulation mechanisms^17–20^. Operons encoding enzymes involved in the degradation of xenobiotics can be located either in plasmids or in the chromosomes. Examples of plasmid operons are those encoding enzymes involved in the degradation of naphthalene, toluene, or xylene, described in pseudomonads (e.g., NAH7, pDTG1^14,17,18^, pWWO^13,19^). In opposition, the operon involved in 2,4,6-trichlorophenol catabolism (*tcp*ABC genes) is located in the chromosome, as suggested by the ability of *R. eutropha* JMP134 (pJP4) and of *R. eutropha* JMP222 (pJP4-cured JMP134 derivative) to degrade this pollutant^21^. Although primarily described as chromosomal^20,22^, the operon involved in biphenyl catabolism (*bph*ABC genes) in pseudomonads, and other distant related *Betaproteobacteria*, is carried by an integrative conjugative element, that can integrate the chromosome and/or plasmid of the host^23^. In addition, genes involved in the same catabolic pathway are not always organized in catabolic operons and can be located in different regions of the chromosome^24^, or sometimes in the chromosome and extrachromosomal replicons (e.g., plasmids). An example of complementarity between chromosome and plasmid genes encoding xenobiotic degradation is given for carbaryl (1-naphthyl N-methylcarbamate) by *Arthrobacter* sp. strain RC100^25^.

This study aimed at a genomic characterization of *G. molinativorax* ON4^T^, particularly at identifying the genes coding for the potential enzymes involved in the ACA degradation pathway. Based on the premise that the genes of this pathway would be in a polycistronic operon unit, we started by searching the *mol*A genetic environment in the publicly available draft genome of *G. molinativorax* DSM13485^T^ (= LMG 21909^T^ = CCUG 49965^T^ = CIP 108515^T^ = ON4^T^) generated by the DOE Joint Genome Institute (JGI, GenBank with the accession number AUDX00000000.1). However, it did not report the *mol*A gene. We obtained the complete genome sequence of strain ON4^T^ and, for comparison purposes, the genome of a variant of *G. molinativorax* ON4^T^ unable to degrade molinate (strain ON4^(−)^, obtained by successively growing in a culture medium without the herbicide for 12 generations). As the genomic data did not reveal other catabolic genes potentially involved in ACA degradation in the vicinity of the *mol*A gene, transcriptomic analyses were further used to identify these genes. Transcripts obtained when strain ON4^T^ grew in the presence and absence of the herbicide were compared and thoroughly analysed. We have identified a novel mobilizable plasmid and seven candidate enzymes involved in ACA mineralization. The future characterization of their in vivo activity will be of great value not only for fundamental microbiology, but to other emerging fields such as biocatalysis and synthetic biology.

## Results and discussion

### Strain ON4^T^ genome sequencing

**Figure 1:**
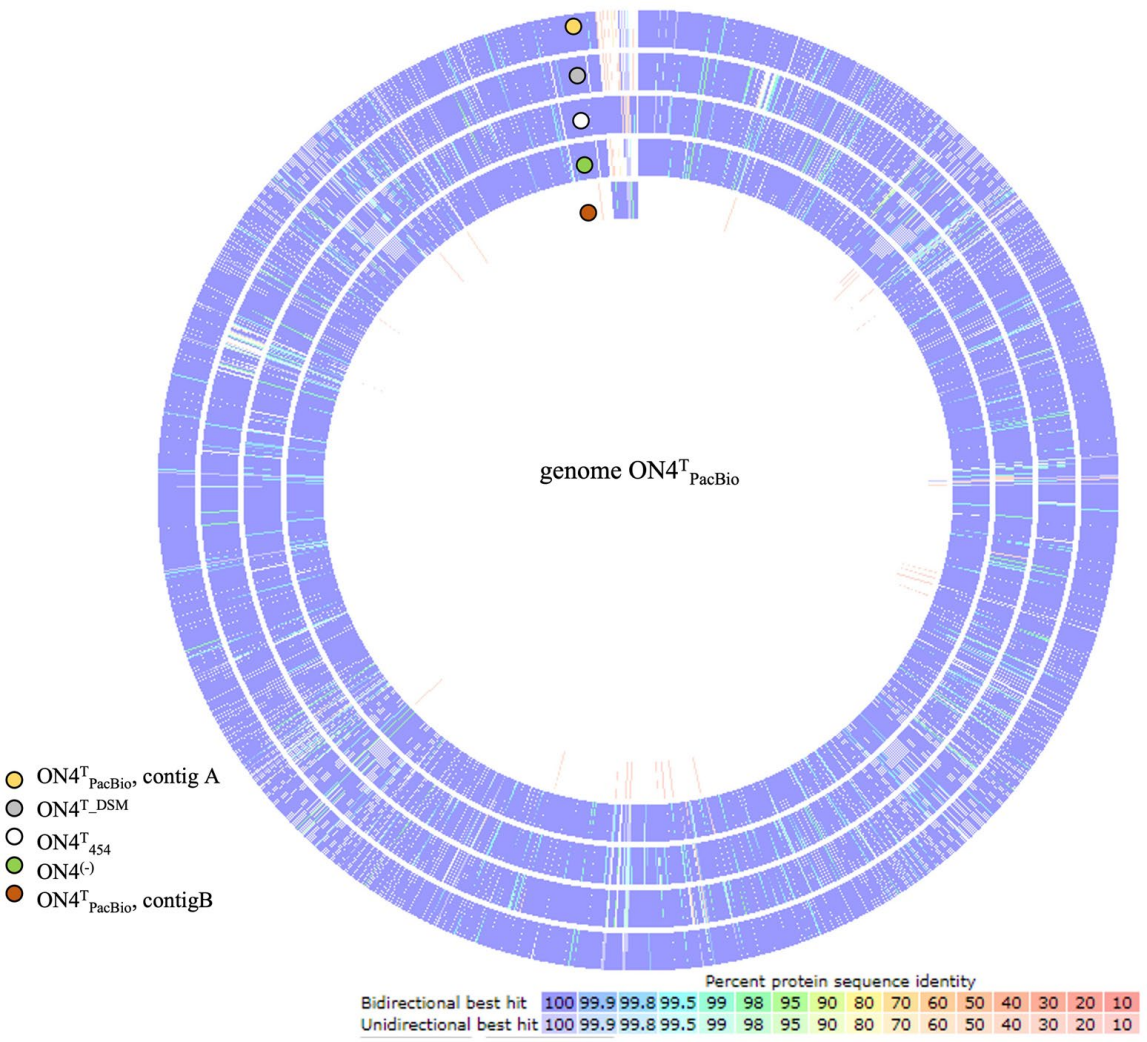
Sequence-based comparison of the reference ON4^T^_PacBio_ (CP028426 and CP028427) with each of the assembled genomes of *G. molinativorax* strains (ON4^T^_454_—PXVE00000000, DSM13485^T^ (= ON4^T^) AUDX00000000.1 and ON4^(−)^—PXVD00000000) using RAST.

The 454 pyrosequencing of the *G. molinativorax* ON4^T^ genome (ON4^T^_454_) generated 305,068 reads, comprising 133.7 million nucleotides (nt), which were assembled in 163 contigs (PXVE00000000). Interested in disclosing the genes in the vicinity of *mol*A, analyses started with a BLAST search of the *mol*A gene sequence against the 163 assembled contigs. The complete *mol*A gene sequence (1398 bp) was located in a single contig (1957 bp) but revealed no other open reading frame (ORF) in its vicinity. The strain ON4^T^ genome was resequenced using PacBio (ON4^T^_PacBio_), which generated 102,855 polymerase reads, resulting in 184,306 subreads with a mean subread length of 7254 bp, and 1,337 billion nt. These reads were de novo assembled and two contigs of 3,465,658 bp (linear) (contig A—CP028426) and 37,013 bp (circular) (contig B—CP028427) were generated. The assembled genome had 98.6% completeness, 0.6% contamination (Table S1) and 64% average G+C content for each contig. This in silico G+C content value agrees with previously published data^7^. The ON4^T^_PacBio_ genome assembly disclosed 3243 predicted protein-coding genes, most of them assigned to putative functions (75.9%) and matching 94.3% of the predicted protein-coding genes in the assembly of strain DSM13485^T^ (JGI, AUDX00000000.1) (Table 1, Fig. 1, Table S1). Furthermore, forty-nine tRNAs, three 16S rRNA, three 23S rRNA, and three 5S rRNA genes were annotated (Table 1). The occurrence of three 16S rRNA gene copies was further confirmed by determining the ratio between the 16S rRNA and the *recA* genes (single-copy gene) in both strains ON4^T^ and ON4^(−)^ (Table S2).

**Table 1:** Strain ON4^T^_PacBio_ genome assembly and annotation metrics.

Feature/attribute	Value
Genome size (bp)	3,502,671
G+C content (%)	64
DNA coding region (bp)	3,152,404
Total genes (No.)	3301
Total contig number (No.)	2
**Contig A** (CP028426)	3,465,658 bp
5S rRNA	3
16S rRNA	3
23S rRNA	3
tRNA	49
Protein-coding genes	3196
Proteins with function prediction	2459
Proteins with gene name	2347
Proteins with enzyme (EC number)	1390
Proteins with GO terms	2406
Proteins assigned to a pathway	277
**Contig B (**CP028427)	37,013 bp
Protein-coding genes	47
Proteins with function prediction	27
Proteins with gene name	20
Proteins with enzyme (EC number)	7
Proteins with GO terms	23
Proteins assigned to a pathway	0

As expected, the data retrieved using both sequencing technologies (454 and PacBio) were very similar (Fig. 1). Nevertheless, the long reads obtained by PacBio sequencing allowed to sum up the 163 assembled ON4^T^_454_ contigs into two contigs only. In addition, the comparison of the two draft genomes allowed closing the contig A (Fig. S1) and identifying the *mol*A gene in the contig B.

### *mol*A is the passenger gene of Tn*6311*

**Figure 2:**
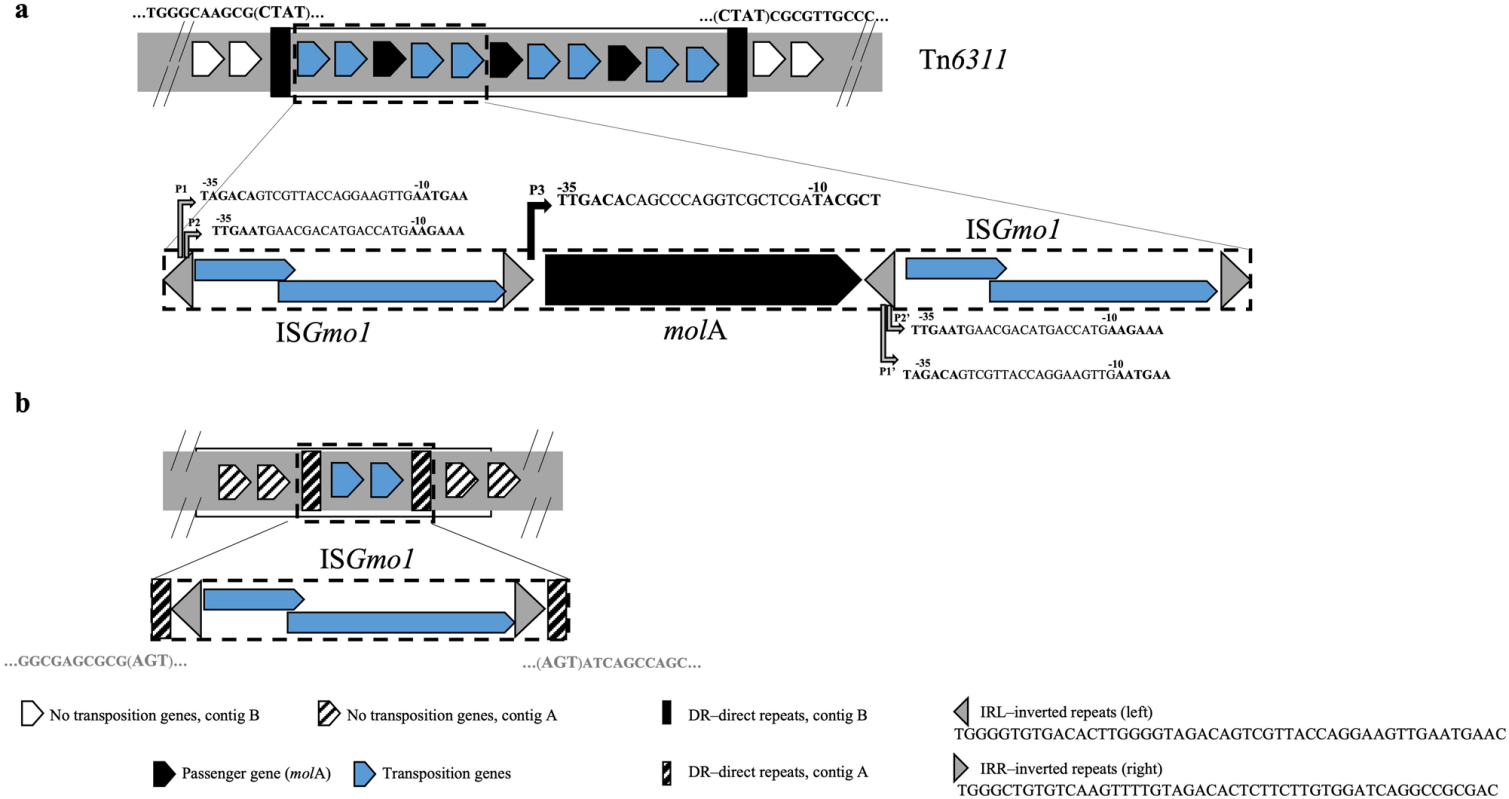
Mapping of the *molA* gene genetic vicinity. (**a**) Representation of the composite transposon Tn*6311* in strain ON4^T^ (ON4^T^_PacBio_—CP028427), as well as the in silico predicted promoters (P) close to *mol*A gene. (**b**) Schematic representation of the IS*Gmo1* without passenger gene in strain ON4^T^ and ON4^(−)^ (ON4^T^_PacBio_—CP028426; and ON4^T^_454_—PXVE00000000; DSM13485^T^ (= ON4^T^)—AUDX00000000.1 and ON4^(−)^—PXVD00000000, http://www.ncbi.nlm.nih.gov/).

The *mol*A gene, located in contig B, was surrounded by two identical insertion sequences (IS) of the IS*3* group and family (Fig. 2a), whose original transposase belongs to the DDE transposase family^26^. The primary sequence of this IS was not identical to any IS described so far and was named IS*Gmo1* (https://www-is.biotoul.fr/). This structure, where a passenger gene (*mol*A), which does not code for a transposition enzyme, is flanked by two IS (IS*Gmo1*), is typical of a composite transposon^27,28^. This organization was confirmed by Sanger sequencing (data not shown). The novel composite transposon, named Tn*6311*, contained three copies of *mol*A, each flanked upstream and downstream by IS*Gmo1* (a total of 4 IS*Gmo1* copies) (Fig. 2a). This tandem organization was primarily observed in specific PacBio raw reads (data not shown). Further, the three *mol*A copies were confirmed in strain ON4^T^ using quantitative PCR, as indicated by the gene copy ratio between *mol*A and a neighbour single-copy gene (*par*A) (Table S2).

As described for other IS*3*-like sequences, IS*Gmo1* has a total length of 1287 bp and contains two partially overlapping open reading frames, *orf*A (291 bp) and *orf*B (1080 bp), which encode the DNA-binding and the catalytic domain of the transposase, respectively^27,29–31^. Some IS*3* family members might assemble different functional protein domains from one DNA segment, using a mechanism known as recoding programmed ribosomal frameshifting. This mechanism involves a ribosomal slippage of 1 nt, occurring at the heptanucleotide sequence A AAA AAG position, between *orf*A and the downstream fragment, *orf*B^26,31^. The slippage generates a −1 frameshift resulting in a fused product of the upstream frame (DNA-binding domain) and the downstream frame (catalytic domain), ORFAB^26,31^. Thus, ORFAB (396 aa) from IS*Gmo1* is a putative protein predicted in silico^32^ based on this mechanism. The three proteins of IS*Gmo1*, ORFA, ORFB, and ORFAB, shared the highest amino acid sequence identity with the respective open reading frames of IS*Fsp8* (45–49%) found in *Frankia* sp. and IS*Aar25* and IS*Aar4* (43–45% and 44–46%, respectively) both found in *Arthrobacter arilaitensis,* also belonging to the phylum *Actinobacteria* (Fig. S2). Although DDE transposases have a highly conserved catalytic structure, they show overall low sequence conservation^33^, which may explain the low identities found between the IS*Gmo1* transposase and those found in the ISfinder database (https://www-is.biotoul.fr/). Despite the low identities, IS*Gmo1* clusters with IS sequences of other *Actinobacteria*, suggesting a phylogenetic relationship (Fig. S2).

In Tn*6311*, the inverted repeats on the left side (IRL) includes the start codon of the *orfA/orfAB* IS*Gmo1* genes and also two putative transposase promoters according to in silico identification (http://genome2d.molgenrug.nl/) (Fig. 2a). In contrast, the repeats on the right side (IRR) of IS*Gmo1*, just upstream the *mol*A gene, includes the last three codons of *orfB* and the − 35 region of the *mol*A promoter. The structure of the *mol*A gene regulatory region, i.e., the sequence of the regions − 35, − 10, and the ribosome binding sequence and their relative position towards the *mol*A start codon (Fig. S3), suggests a strong promoter^15,34^. The existence of such a strong promoter combined with the tandem organization of *mol*A gene in Tn*6311* explains the high rate of molinate breakdown by strain ON4^T^, suggesting a high gene expression response.

Interestingly, the IS*Gmo1* sequence was also found in contig A (ON4^T^_PacBio_), but without any passenger gene (Fig. 2b) and with different direct repeats sequences. Evidence of such a copy of IS*Gmo1* was also given by the ON4^T^_454,_ DSM13485^T^ (JGI, AUDX00000000.1), and ON4^(−)^ sequenced genomes and confirmed by primer design and Sanger sequencing (data not shown).

### Characterization of contig B: pARLON1

Contigs A and B were presumed, based on size, to be a chromosome and a plasmid, respectively. The detection of a band with approximately 30 kbp by Pulsed-Field Gel Electrophoresis (PFGE) (Fig. S4) supported this hypothesis. Moreover, no reads mapping to contig B were found in the draft genomes of strains DSM13485^T^ (JGI, AUDX00000000.1) and ON4^(−)^ (Fig. 1), an ON4^T^ variant unable to degrade molinate (Fig. S5). Altogether these data corroborate the existence of a mobile element enabling the degradation of molinate, i.e., a plasmid carrying the *mol*A gene, which, most probably, was cured in strain ON4^(−)^ after 12 generations in culture medium without the herbicide. In turn, the absence of reads mapping to contig B in strain DSM13485^T^ (JGI, AUDX00000000.1) suggests that the conditions used to grow the culture collection strain did not favour the maintenance of the plasmid that is quite unstable. Hence, contig B was named pARLON1.

**Figure 3:**
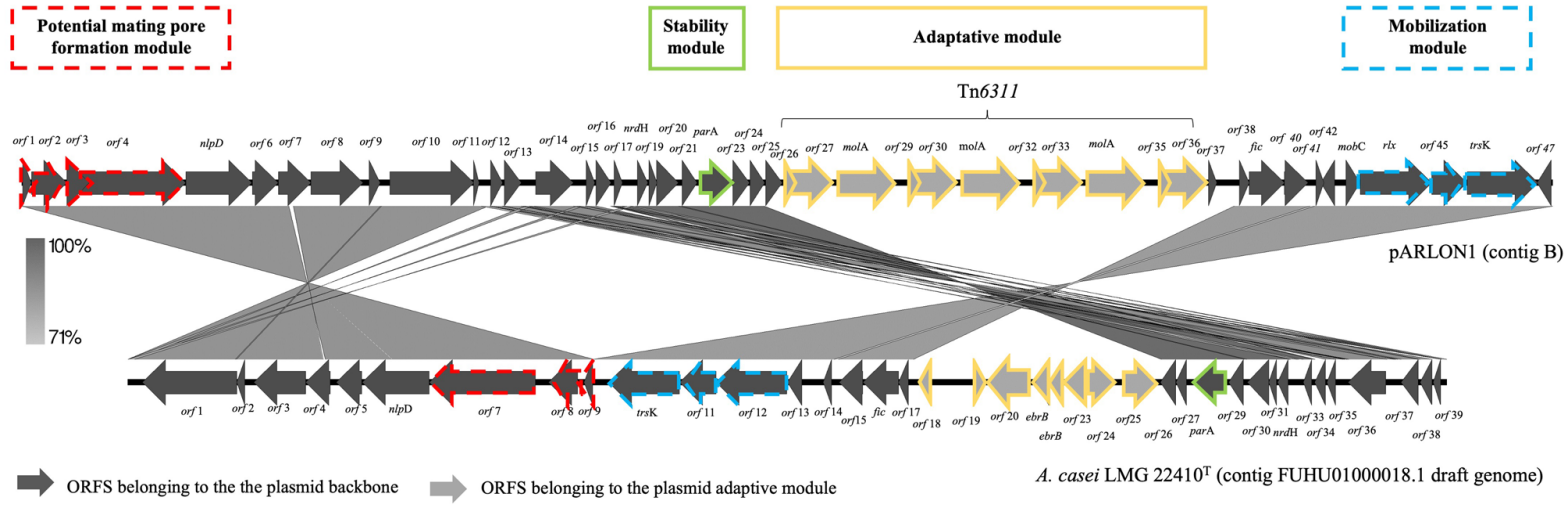
Genetic organization of pARLON1 and scheme of the BLASTn search results between pARLON1 (contig B) and *A. casei* LMG 22410^T^ (contig FUHU01000018.1 draft genome) using Easyfig software^39^. Plasmid genes predicted by CONJscan^40^ and/or oriTDB^41^ as related to a putative T4SS of the type_FATA_ are represented by dash lines.

Plasmids are described as having modular structures, where each “module” is dedicated to a particular function (replication, stability, conjugation, establishment and adaption)^35^. pARLON1 contained 47 identified ORFs from which 36 ORFs (~ 27 kbp) belong to the plasmid backbone, and 11 ORFs belong to the adaptive module (Tn*6311*, ~ 10 kbp) (Fig. 3). pARLON1 did not share significant nucleotide identity with other described plasmids (< 70%), not even with non-conjugative plasmids harboured by other *Microbacteriaceae* members (e.g., plasmids pCM1 and pCM2 of *Clavibacter michiganensis* subsp. *michiganensis*, closely related with *G. molinativorax*)^36^. However, pARLON1 shared a high nucleotide identity (88.2%) with the contig 18 of *Agrococcus casei* LMG 22410^T^ draft genome (Contig FUHU01000018.1, GenBank FUHU00000000.1), as determined by ANI^37^ (Table S3). LMG 22410^T^ is the type strain of *Agrococcus casei*, also a member of the family *Microbacteriaceae*, with a G+C content (65 mol%) closer to that of *G. molinativorax* (64 mol%) than to other *Agrococcus* species^7,38^. The pARLON1 genes sharing > 70% identity with genes of the *A. casei* contig were those belonging to the plasmid backbone (36 predicted genes), representing 46.3 and 53.8% of the full coverage of contig B and contig FUHU01000018.1, respectively. In contrast, the predicted genes belonging to the adaptive module of each of these genetic elements were different (Fig. 3, Table S4).

Some ORFs of both pARLON1 and contig FUHU01000018.1 are related to the functional modules of transmissible plasmids, according to the annotation of pARLON1 and the in silico predictions performed by CONJscan^40^ and by oriTDB^41^. These analyses predicted the existence of two essential proteins of the single-strand DNA conjugation machinery, the relaxase (MOB) and the type IV coupling protein (T4CP). The former is a critical component of the relaxosome responsible for initiating the DNA transfer in both conjugative and mobilizable plasmids, and the last couples the relaxase-DNA nucleoprotein complex to the type IV secretion system (T4SS)^42^. A T4SS is a large complex of proteins spanning from the cytoplasm to the cell exterior constituting the mating-pair formation (MPF) module^43,44^. ORF44 and ORF12 from strains ON4^T^ and *A. casei*, respectively, showed homology with MOB proteins, whereas ORF46/*trs*K and ORF10 were related to the TraG/TraD family protein whose members are homologs to T4CP^45,46^ (Fig. 3, Table S4). However, only CONJscan predicted the existence of an ORF related to VirB4 ATPase (ORF4 and ORF7 from strains ON4^T^ and *A. casei*, respectively), a marker of a T4SS presence^43,44^ (Fig. 3, Table S4). Hence, according to CONJscan, both ON4^T^ and *A. casei* harbour a putative T4SS of the class FATA^40^, which is a MPF family specific to ***F****irmicutes, ****A****ctinobacteria*, ***T****enericutes* and ***A****rchaea*^43,44^, agreeing with the taxonomic affiliation of both ON4^T^ and *A. casei*. Because members of these microbial groups lack an outer membrane, some of the core complex T4SS components (e.g., VirB7/10) of *Agrococcus tumefaciens* are not identified in all the well-characterized conjugation systems of MPF_FATA_ type (e.g., pCF10 and pGO1)^44^. However, proteins homologous to VirB3, VirB6 and VirB8, proteins of the inner-membrane pore, are common within this system type^44^. Among these MPF proteins, CONJscan predicted the existence of PrgH in ON4^T^ (ORF2) and *A. casei* (ORF8), which show homology with VirB6^44^. Also, PrgI, which is suggested to have homology with VirB3^44^, was predicted in ON4^T^ (ORF3). In addition, CONJscan predicted the presence of two other ORFs (1 and 45, and 9 and 11 from strains ON4^T^ and *A. casei*, respectively) related to accessory genes detected at frequencies higher than 50% within the MPF_FATA_ family (FATA_*prg*F and FATA_*cd411*). Although these predictions support the existence of a MPF_FATA_ type, the presence of a functional MPF module in pARLON1 needs to be experimentally validated.

In addition, the pARLON1 backbone seems to carry a stability module. This module is particularly important in low copy number plasmids, where mechanisms, such as the partition (Par) systems, are required to ensure the even partitioning of the DNA molecules among progeny (vertical transmission)^35,47,48^. ORF22 and ORF28 from strains ON4^T^ and *A. casei*, respectively, were predicted to be related to the plasmid partition motor protein ParA. However, no homologous to other proteins of this system (e.g., ParB and ParS) were predicted. These results may indicate that the Par system is incomplete/not functional (which could explain the existence of the cured strains DSM13485^T^ and ON4^(−)^), or that the existing DNA binding proteins have low homology to known Par proteins (lower homology was found for proteins of the ParB group than for group ParA, difficulting the classification of the former^49^), or that another maintenance mechanism is present. At the population level, conjugative plasmids are described as low copy number, while mobilizable plasmids tend to be high copy number^35,50^. The ratio between the plasmid (*par*A) and chromosomal (*recA)* single-copy genes in strain ON4^T^ was 3:1 (Table S2), suggesting that pARLON1 is a low copy number plasmid^51^. The presence of ORFs potentially associated with the maintenance and conjugation modules, together with the plasmid size (> 30 kbp) and its low copy number, suggest that pARLON1 can be a conjugative plasmid^35^. However, further studies are necessary to validate its conjugative activity.

As referred to above, the adaptive module of pARLON1 differed from that of contig 18 from strain *A. casei* LMG 22410^T^*.* However, the similarity of the plasmid backbone and the identification of a putative MPF_FATA_ in strains ON4^T^ and *A. casei* LMG 22410^T^ strengthens the existence of a common actinobacterial ancestor that, depending on the environment, acquired different adaptive modules. Moreover, plasmid evolution might have been mediated via the activity of the insertion sequences flanking the adaptive module, IS*6100* in *A. casei* LMG 22410^T^ and IS*Gmo1* in Tn*6311* of *G. molinativorax* ON4^T^.

### Identification of candidate genes involved in ACA mineralization

Given the absence of any other catabolic gene in the vicinity of *mol*A, a transcriptomic approach was used to identify the potential genes involved in ACA mineralization. The gene expression profiles of strain ON4^T^ growing in molinate (mineral medium with the herbicide as the single source of energy, carbon and nitrogen, herein referred to as ON4^T^_MMM_) and without molinate (control medium LB, herein referred to as ON4^T^_LB_) were compared to identify the genes overexpressed in ON4^T^_MMM_.

After quality control, 14,380,135 and 15,330,180 reads in each library (ON4^T^_MMM_ and ON4^T^_LB_, respectively) were retrieved. Analysis of the reads revealed that subtractive hybridization only removed a small amount of the ribosomal RNA, since 86–90% of reads mapped to rRNA. Consequently, approximately 1.4 and 1.0 million mRNA sequence reads were obtained from ON4^T^_MMM_ and ON4^T^_LB_, respectively. After mapping the mRNA reads against the ON4^T^ genome sequence, 95% of the 3243 expected transcripts were expressed under the tested growth conditions (ON4^T^_MMM_ and/or ON4^T^_LB_) (Table S5). From the 3095 expressed transcripts, 2726 were expressed under both growth conditions and 335 were overexpressed in ON4^T^_MMM_ compared to ON4^T^_LB_ (Tables S6 and S7)_._ Of these, 149 transcripts were exclusive in ON4^T^_MMM_, i.e., no reads were detected in ON4^T^_LB_. RT-qPCR validated expression results in eight overexpressed genes in ON4^T^_MMM_. The trend of expression level was similar to that of the transcriptomics experiment (Table 2).

**Table 2:** Validation of transcriptomics results by RT-qPCR.

Symbol	Predicted gene product	RNA-Seq data	RT- qPCR data
		LOG2FC^a^	Average_Nrelative fold expression_^b^ ± SE
*mol*A^#^	Molinate hydrolase	15.21	393.27 ± 14.91
		15.21	
		15.21	
*met*E	5-methyltetrahydropteroyltriglutamate-homocysteine methyltransferase	8.80	103.38 ± 11.35
*chn*C	Caprolactone hydrolase	3.56	387.49 ± 78.21
*cin*C	Nitric oxide synthase	5.85	5.40 ± 0.18
*adh*P	Alcohol dehydrogenase	5.85	37.59 ± 2.70
*fpr*A	NADPH-ferredoxin reductase FprA	7.44	6.32 ± 0.20
*hyu*A	Hydantoin utilization protein A	3.59	2.85 ± 0.15
*par*A*	Plasmid partition protein homolog ParA	N.D	579.00 ± 69.16

^a^Fold-change was calculated as the ratio of the expression level of strain ON4^T^ growing in MMM by the expression level of strain ON4^T^ growing in LB. Log2FC > 2 was considered as different and indicative of overexpressed genes in ON4^T^_MMM_. For *par*A gene, as the expression was 0 for ON4^T^_LB_ (i.e., reads mapping this gene were not detected), it was not possible to obtain the FC value (N.D.);

^b^Defined as the ratio of the fold change of each target gene (E_target_^[Ct_LB_-Ct_MMM_]) by the fold change of reference gene (geomean of E_ref_^[ΔCt Ct_LB_-Ct_MMM_], based on the results obtained from each of the 3 reference genes selected in the present study);

^#^There are 3 copies of this gene;

*Ct values obtained for strain ON4^T^ growing in LB are close to 40, the lowest value for qPCR, which corroborates the data from cDNA libraries where the detection of this transcript is only possible in strain ON4^T^growing in MMM.

*N.D.* not detected, *SE* standard error.

Metabolic pathways for *Gulosibacter* sp. are not available in KEGG. Nevertheless, the predicted gene products from the 335 overexpressed transcripts in ON4^T^_MMM_ were mapped to the KEGG database^52^. Some of the overexpressed predicted gene products in ON4^T^_MMM_ mapped to KEGG Orthology (KO) categories (Fig. S6), namely to metabolism (e.g., amino acid, carbohydrates), environmental information processing (e.g., membrane transport) and Brite hierarchies (e.g., signaling and cellular processes), respectively. However, no complete KEGG pathway appeared to be overexpressed in ON4^T^_MMM_, in particular, none that could be related to ACA mineralization. Most of the predicted gene products mapped to more than one metabolic pathway and/or different predicted gene products mapped to the same metabolic function.

**Figure 4:**
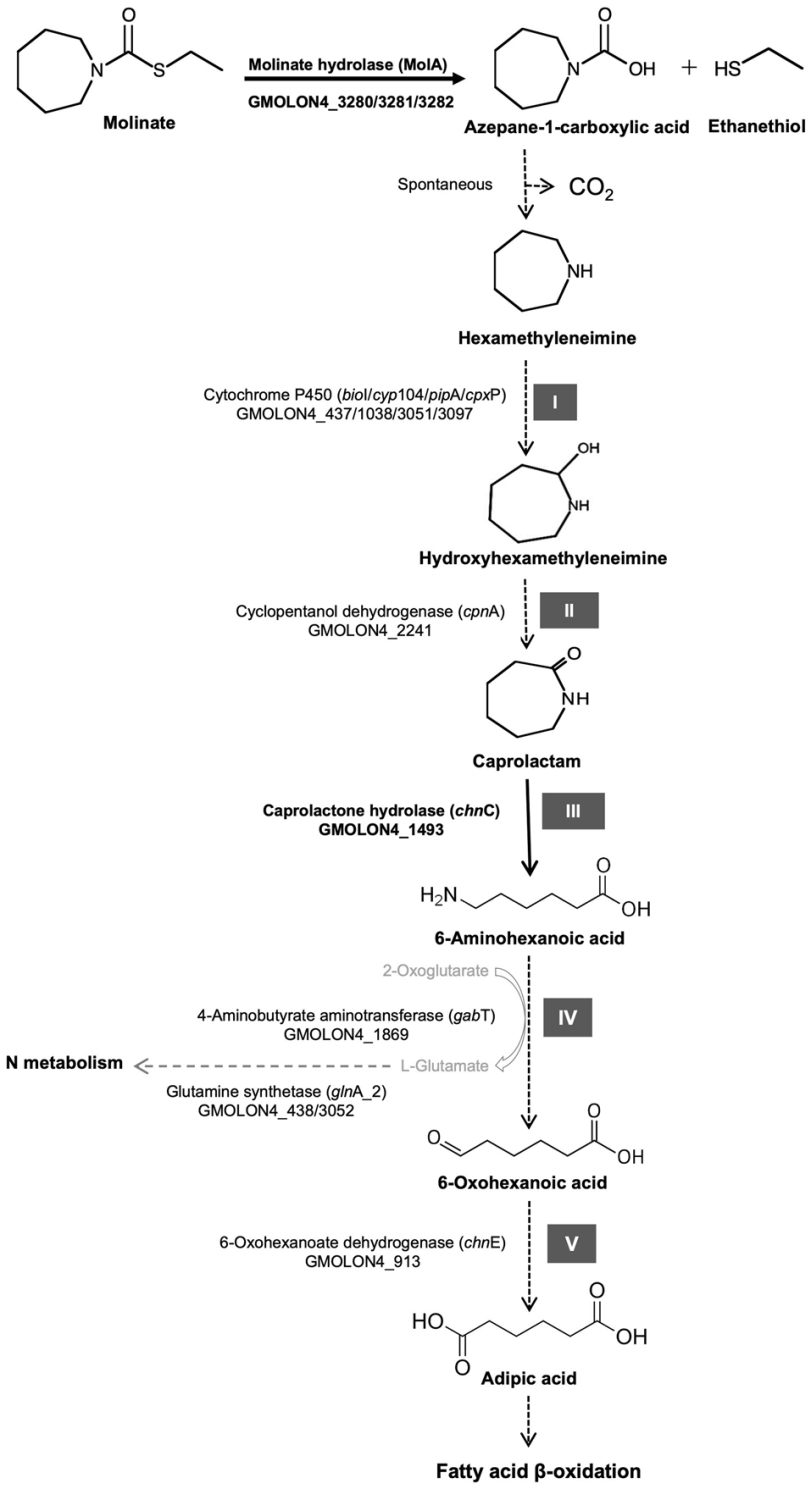
Candidate enzymes involved in the five steps (I–V) of the putative molinate degradation pathway by strain ON4^T^ adapted from Barreiros and collaborators^8^.

Thereby, genes potentially involved in ACA mineralization were identified based on homology to previously described enzymes involved in the degradation of other xenobiotics^53–56^ and on the structural similarity of the putative substrates (Fig. 4). Potential candidates involved in the breakdown of the heterocyclic ring containing metabolites are cytochromes P450 (CYP), which act as monooxygenases and are among the most versatile biological catalyst^57^, being, among others, involved in the degradation of different xenobiotics^57,58^. In typical class I P450 systems, the electrons required for oxygen activation are indirectly provided by NAD(P)H. The co-enzyme generally reduces a ferredoxin reductase (FDR), which in turn transfers the electrons to the second component of the system, usually a ferredoxin^57^. Finally, the ferredoxin mediates the electron transport between the FDR and the CYP^57^, further inducing the incorporation of one oxygen atom into the substrate via the reductive cleavage of oxygen.

**Figure 5:**
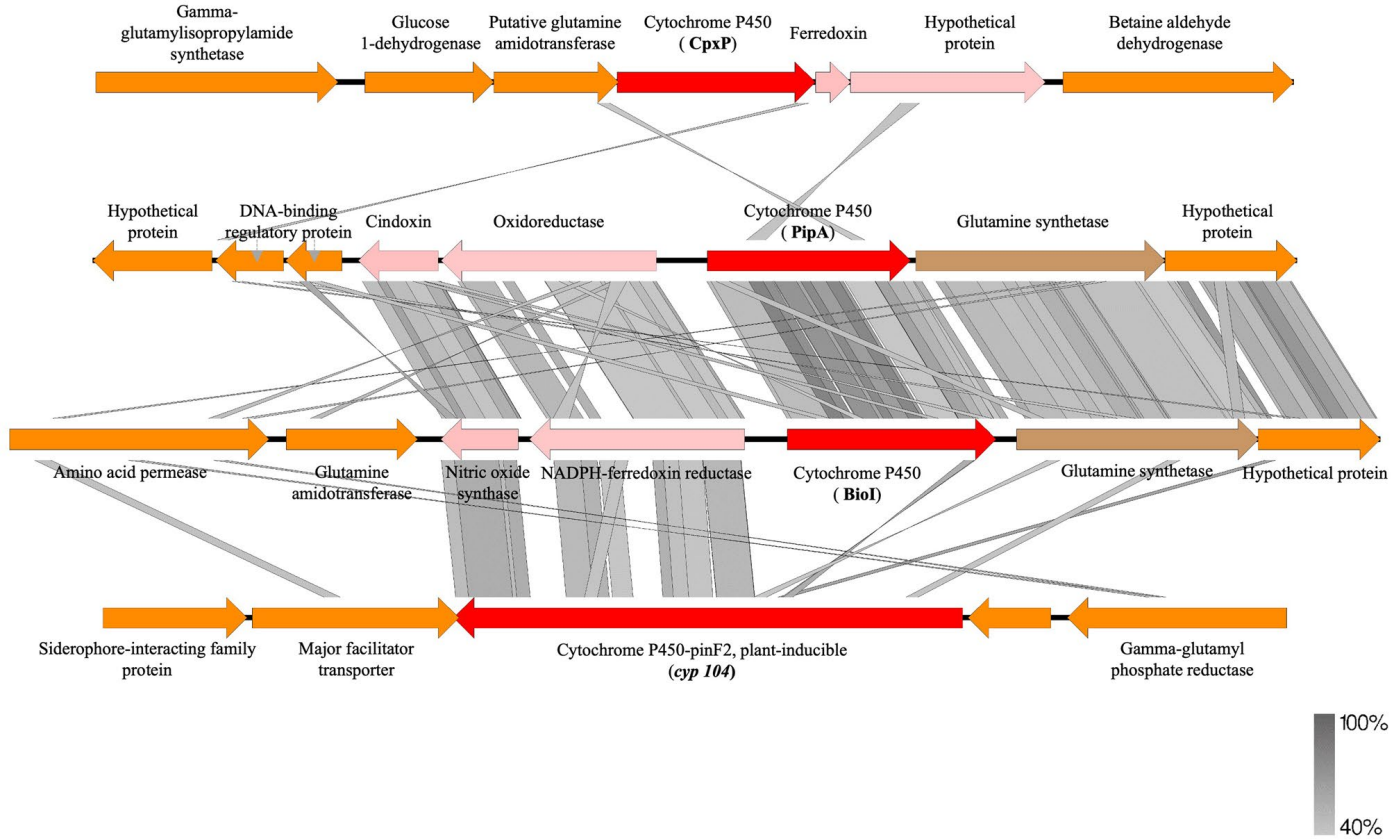
BLASTx between genes in the vicinity of cytochrome P450 (*cpx*P—GMOLON4_3097, *pip*A—GMOLON4_3051, *bio*I—GMOLON4_437 and *cyp*104—GMOLON4_1038, respectively) from strain ON4^T^ (Accession Number CP028426).

Strain ON4^T^ harbours four ORFs with homology to cytochromes P450, namely *bio*I, *cyp*104*, pip*A and *cpx*P (Fig. 5, Table S8)*.* Among these, *bio*I and its contiguous ferredoxin reductase (GMOLON4_437 and GMOLON4_436, respectively) were highly expressed in ON4^T^_MMM_ (Log2FC > 6, Table S8). Also, *cyp*104 (GMOLON4_1038) and *cpx*P (GMOLON4_3097) were overexpressed in ON4^T^_MMM_ (Log2FC > 3, Table S8), whereas *pip*A and its contiguous ferredoxin reductase (GMOLON4_3051 and GMOLON4_3050, respectively) were not significantly overexpressed in ON4^T^_MMM_ (Log2FC < 2, Table S8). Noticeably, the cytochromes P450 *bio*I and *pip*A of strain ON4^T^ have an amino acid identity of 72% and have a similar genetic neighbourhood (Fig. 5). Indeed, the upstream and downstream enzymes gene sequences seem to be similar, particularly the contiguous ferredoxin reductase GMOLON4_436 and GMOLON4_3050, respectively, which have an amino acid identity of 48% with each other. Also, the proteins potentially involved in the transfer of the electrons from the ferredoxin reductases to the cytochromes P450 *bio*I and *pip*A (GMOLON4_435 and GMOLON4_3049, respectively) share an amino acid identity of 69%. In contrast, these cytochromes (*bio*I and *pip*A) share less than 26% and 30% amino acid identity with the cytochromes *cyp*104 and *cpx*P, respectively, which are also distinct from each other (24% amino acid identity) (Fig. 5).

Curiously, the cytochromes P450 *bio*I and *pip*A of strain ON4^T^ have an amino acid identity of approximately 53 and 52% with that of *Mycobacterium smegmatis* mc^2^155 (*pip*A, AAD28344.1) and *Mycobacterium* sp. HE5 (*mor*A, AAV54064.1), respectively (Table S8). Both are shown to be involved in the hydroxylation of N-heterocycles^53,59^. *M. smegmatis pip*A was shown to be induced when strain mc^2^155 grew in piperidine as the sole source of energy, carbon, and nitrogen^53^. *Mycobacterium* sp. HE5 recombinant protein *mor*A in combination with the adjacent pair ferredoxin reductase/ferredoxin was able to oxidize morpholine^59^. Although the contiguous *bio*I and *pip*A ferredoxin reductase (GMOLON4_436 and GMOLON4_3050, respectively) were distinct from those found in *Mycobacterium* strains (Fig. S7), the inhibition of ACA mineralization under anaerobic conditions^8^, combined with the similarity between the molecular structure of piperidine/morpholine (six/five membered N-heterocycles), and the predicted seven membered N-heterocycle product from ACA degradation in strain ON4^T^ (hexamethyleneimine)^8^ (Fig. S7), supports the involvement of one of these systems in the hydroxylation of hexamethyleneimine to hydroxyhexamethyleneimine in strain ON4^T^^8^ (step I, Fig. 4). However, further experimental validation is necessary.

According to previous studies, the resulting hydroxyhexamethyleneimine is further oxidized to caprolactam^8^, which suggests the activity of a dehydrogenase (step II, Fig. 4). Cyclopentanol dehydrogenase (*cpn*A, GMOLON4_2241) is among the overexpressed dehydrogenases in ON4^T^_MMM_ (Log2FC > 2, Table S8). It shows 40% amino acid identity with cyclopentanol dehydrogenases from *Comamonas* sp. NCIMB 9872^60^ and 31–34% identity with cyclohexanol dehydrogenases from *Rhodococcus* sp. TK6^61^ and *Acinetobacter johnsonii*^62^, respectively, known to oxidise cyclic alcohols but not N-heterocyclic alcohols, such as hydroxyhexamethyleneimine^63–65^.Therefore, the role of the GMOLON4_2241 product in the degradation of molinate in strain ON4^T^ needs further experimental validation.

Considering the further step of the putative degradation of molinate, the cleavage of the cyclic metabolite (caprolactam) into 6-aminohexanoic acid (step III, Fig. 4), two possibilities arose from the transcriptomics analysis. These were the gene products of *hyu*A, GMOLON4_3203, and *hyu*B, GMOLON4_3204 (hydantoin utilization protein A and hydantoinase B, respectively), and the gene product of *chn*C (GMOLON4_1493, caprolactone hydrolase). All were overexpressed in ON4^T^_MMM_ (Log2FC > 2, Table S6). GMOLON4_3203 and GMOLON4_3204 showed the highest amino acid identity (72–90%) with proteins of the Hydantoinase-Hydantoinase B/Oxoprolinase family found in different members of the phylum *Actinobacteria*. Whereas GMOLON4_1493 showed the highest amino acid identity (75–79%) with *Alpha/Beta* hydrolases, followed by caprolactone hydrolase (72–75%) of *Rhodococcus* sp. strains HI-31 and Phi2^66,67^. Both hydantoinases/oxoprolinases and caprolactone hydrolase are described as able to open cyclic organic compounds, by hydrolising C-N bonds in cyclic amides and opening the lactone ring, respectively.

**Figure 6:**
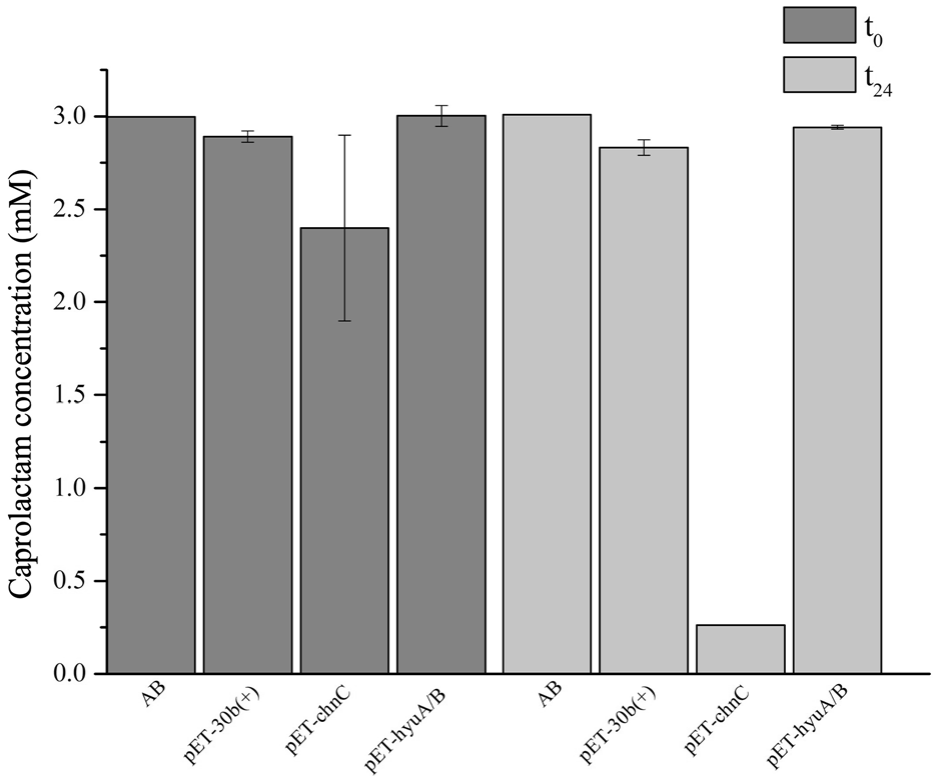
Evaluation of caprolactam degradation in resting cells assays (DOλ_600nm_ ≈ 6), by *E. coli* BL21(DE3) strain carrying either pET-30b( +), pET-*chn*C or pET-*hyu*A/B over a 24 h incubation period in 50 mM of phosphate buffer (pH 7.2) and 3 mM caprolactam. Values are means ± SE (n = 3, for all biological assays). AB—abiotic control.

Caprolactam is the raw material for manufacturing Nylon-6 by ring opening polymerization. Attempts to isolate organisms capable of attenuating the environmental pollution caused by caprolactam intensive production and utilization have been made. Up to now, few Gram negative and Gram positive bacterial strains, including *Gulosibacter* sp. BS4, were described as capable of caprolactam degradation^56,68–70^. Among these organisms, only the enzyme acting upon the cleavage of caprolactam in *Pseudomonas jessenii* GO3 was described^56,71^. This caprolactamase, which consists of two subunits with high sequence identity to 5-oxoprolinases, hydrolyzes caprolactam into 6-aminohexanoic acid in an ATP-dependent manner^56,71^. Such information suggested the possible involvement of the GMOLON4_3203 and GMOLON4_3204 gene products in the breakdown of caprolactam by strain ON4^T^. However, heterologous expression of both potential candidates demonstrated that only the *chn*C gene product (GMOLON4_1493) degraded caprolactam in resting cells assays (Fig. 6). Proteins affiliated to this family are involved in the cyclic ε-caprolactone transformation into a linear organic acid, formed during the oxidation of cyclohexanol or cyclohexanone by both Gram staining positive and negative bacteria^54,55,66,67^. Hence, as far as we know, this is the first report of their involvement in caprolactam degradation.

Although only 6-oxohexanoate dehydrogenase (GMOLON4_913, *chn*E) was overexpressed in ON4^T^_MMM_ (Log2FC > 3, Table S8), both 4-aminobutyrate aminotransferase (GMOLON4_1869, *gab*T) and 6-oxohexanoate dehydrogenase (GMOLON4_913, *chn*E) are potentially involved in the transformation of 6-aminohexanoic acid to adipic acid (step IV and V, Fig. 4). Indeed, the 4-aminobutyrate aminotransferase (GMOLON4_1869, *gab*T) and the 6-oxohexanoate dehydrogenase (GMOLON4_913, *chn*E) have an amino acid identity of 57 and 80% with the NylD1 6-aminohexanoate aminotransferase and the NylE1 adipate semialdehyde dehydrogenase from the Nylon oligomer-degrading actinobacterium *Arthrobacter* sp. strain KI72^24^, respectively (Table S8). In strain KI72, NylD1 in combination with the cofactor pyridoxal phosphate (PLP) catalyse the transference of the amino group from 6-aminohexanoate to an amino acceptor (e.g., α-ketoglutarate, pyruvate, and glyoxylate) generating 6-oxohexanoate and an amino-acid (e.g., glutamate, alanine, and glycine), respectively. Then, NylE1 catalyses the oxidation of 6-oxohexanoate to adipate using NADP^+^ as a cofactor, enabling the *Arthrobacter* sp. strain KI72 growth on 6-aminohexanoate as a sole source of carbon and nitrogen^24^. Similarly, the adipic acid formed through the activity of the 6-oxohexanoate dehydrogenase (GMOLON4_913, *chn*E) may enter the fatty acid β-oxidation providing further energy for strain ON4^T^ metabolic activity^72^ (Fig. 4). Indeed, the enzymes of this central metabolic pathway were overexpressed in ON4^T^_MMM_ (Table S8). The central metabolism activated via succinyl-CoA and acetyl-CoA from fatty acid β-oxidation may be thus related to the ability of strain ON4^T^ to use ACA as the single source of carbon, nitrogen, and energy^8^.

Also, the overexpression of transcripts related to the metabolism of N-compounds, particularly glutamine synthetase GMOLON4_438/3052 (Fig. 4 and Fig. S6, Table S8), agrees with the ability of strain ON4^T^ to grow on molinate as the single source of nitrogen^8^. The importance of glutamine synthetase and glutamate synthase cyclic mechanism in ammonium assimilation in prokaryotes is well known. In particular, Harper and collaborators^73^ reported that either glutamate dehydrogenase or glutamine synthase are very responsive and regulated under high or low nitrogen availability in *M. smegmatis*.

Among the proteins potentially involved in molinate degradation, only those related with the cytochromes P450 BioI and PipA seem to be found in some strains of the genus *Gulosibacter* (amino acid identity of up to 72% and 91% respectively, Table S9). These results suggest that some *Gulosibacter* strains, such as *G. chungangensis* KCTC 13959^T^, may have the ability to partially oxidize N-heterocycle compounds.

Interestingly, the products of the genes putatively involved on hexamethyleneimine degradation in strain ON4^T^ showed relatively high amino acid identity with proteins involved in the oxidation of 6 membered N-heterocycle compounds (piperidine) and cycloalkane alcohols (cyclopentanol/cyclohexanol) by a wide diversity of strains, namely actinobacteria (Table S8). All these results suggest that small alterations on the structure of these proteins may have allowed the utilization of caprolactam in strains ON4^T^ and *Gulosibacter* sp. BS4 and ACA in strain ON4^T^ as energy, carbon, and nitrogen sources.

## Conclusions

In summary, the present study, allowed the in silico identification and characterization of a novel transposon (Tn*6311*), which harbours *mol*A, the enzyme responsible for the breakdown of the molinate to ACA and confers the unique degrading ability of *G. molinativorax* ON4^T^. In addition, a novel transmissible plasmid (pARLON1), which is present in strain ON4^T^ and *Agrococcus casei* LMG 22410^T^, was characterized in silico*.* This last finding is of particular importance as the characterization of actinobacterial transmissible plasmids (mobilizable or conjugative) is scarce in the literature, compared to other bacterial phyla, particularly *Proteobacteria*. The future characterization of the plasmid transmission pathway will enlighten the underrepresented type IV secretion system (T4SS) in the FATA group, which includes only 3 representatives^44^. Such knowledge would enrich databases such as CONJscan^40^ and oriTDB^41^, contributing to better identifying this system, particularly in genomic mining studies.

Additionally, this study identified seven candidate genes involved in ACA mineralization, and validated the activity of the GMOLON4_1493 product, related to the caprolactone hydrolase *chn*C, in the degradation of caprolactam. The future characterization of these enzymes will contribute to enlightening the molinate degradation pathway in strain ON4^T^ and strengthening their biocatalytic potential.

## Material and methods

### Culture conditions

*Gulosibacter molinativorax* ON4^T^ and the variant strain ON4^(−)^ were preserved in 15% (v/v) glycerol suspensions at − 80°C^7^. Whenever necessary, stored cells were recovered in Luria–Bertani Agar supplemented with 1 mM molinate (LAM). Cultures were incubated at 30 °C for 72 h. For 454 pyrosequencing, strain ON4^T^ was grown on LAM, and for PacBio sequencing in mineral medium B^9^ supplemented with 0.2 g/L yeast extract and 1.5 mM molinate (MMM). For Illumina sequencing, strain ON4^(−)^ was grown in LAM. For a detailed description see the Supplementary Material.

### Genome analysis (sequencing, assembling and annotation)

454 pyrosequencing and PacBio technologies were used to sequence the genome of strain ON4^T^. For 454 pyrosequencing, five hundred nanograms of high-quality genomic DNA were fragmented by nebulization and the sequencing adapters ligated to create double-stranded DNA libraries. The 454 GS FLX sequencing platform (Roche) at Genoinseq (Cantanhede, Portugal) was used following the standard manufacturer’s protocols (see Supplementary Material). Sequencing reads were quality trimmed and de novo assembled by GS Assembler, version 2.9 (Roche) using default parameters.

Genome resequencing was performed using SMRT technology PacBio RSII on the RS sequencer at GATC Biotech AG (Constance, Germany), according to the manufacturer’s instructions^74^. Genome assembly was performed on the SMRT analysis software (http://www.pacb.com/products-and-services/analytical-software/smrt-analysis/). The Hierarchical Genome Assembly Process (HGAP, RS HGAP Assembly.3), which includes pre-assembly, de novo assembly with PacBio’s AssembleUnitig and assembly polishing with Quiver, was used with the default parameters, except for the minimum polymerase read quality, where 0.80 was used instead of 0.75. Gene prediction was carried out with GeneMarkS^75^ using specific parameters for prokaryotes (genetic code 11) and functional annotation was performed using the Sma3s^76^. The completeness and contamination of the SMRT assembled genome of strain ON4^T^ were calculated with CheckM^77^.

For the draft genome of strain ON4^(-)^ a DNA library was prepared from one nanogram of high-quality genomic DNA with the Nextera XT DNA Sample Preparation Kit (Illumina, San Diego, USA), which was sequenced using paired-end (PE) 2 × 300 bp on the MiSeq^®^ Illumina^®^ platform at Genoinseq (Cantanhede, Portugal). All procedures were performed according to the standard manufacturer’s protocols. Following demultiplex and quality-filter procedures, the high quality and adapter free reads were assembled with SPAdes, version 3.7.1^78^, using in-house defined parameters.

### Confirmation of the *mol*A regulatory region

The 5′-Rapid Amplification of cDNA Ends (5′ RACE) was performed to identify the *mol*A gene regulatory region using RNA extracted from strain ON4^T^ grown in MMM. For a detailed description see the Supplementary Material.

### Comparison of different assemblies

The assemblies obtained from all the *G. molinativorax* genome sequencing projects (PRJNA245014 present study and PRJNA188907) were compared using MAUVE software^79^ and the RAST server^80^. In addition, the raw reads of genomes ON4^T^_454_ (SRR6825088) and ON4^(−)^ (SRR6825089) were also mapped against the contigs obtained in SMRT analysis software (genome ON4^T^_PacBio_) (https://www.pacb.com/products-and-services/analytical-software/smrt-analysis/), using the BWA-MEM algorithm^81^. The alignments were evaluated using Qualimap v2.2.1 software^82^ and were visualized in Tablet software^83^.

A BLASTx search of contig B against *Agrococcus casei* LMG 22410^T^ draft genome (PRJEB19012) was performed (https://blast.ncbi.nlm.nih.gov/). In addition, the comparison (through BLASTn) between contig B and contig FUHU01000018.1 of *Agrococcus casei* LMG 22410^T^ was carried out using the Contiguity software^84^, and their similarity was measured by the Average Nucleotide Identity (ANI) calculator (https://www.ezbiocloud.net/tools/ani).

### Growth conditions, RNA extraction and sequencing of cDNA libraries

Given the low biomass yield of strain ON4^T^ in mineral medium supplemented with a single carbon source other than molinate, strain ON4^T^ was grown up to the exponential phase in MMM (ON4^T^_MMM_) and Luria–Bertani Broth (ON4^T^_LB_) in 250 mL Erlenmeyer flasks to obtain mRNA libraries. Total RNA was extracted using the RNeasy extraction kit (QIAGEN GmbH) (detailed protocol in the Supplementary Material). The RNA quality was assessed using the RNA 6000 Pico Kit with the Prokaryote Total RNA Pico assay (Agilent 2100 Agilent Technologies, Santa Clara, CA, USA).

The subtractive hybridization protocol described by Stewart et al.^85^ was used (detailed protocol in the Supplementary Material). The resulting rRNA-subtracted products were used to build the cDNA libraries for strand-specific RNA sequencing. The library preparation followed the protocol for whole transcription libraries in the Ion total RNA-Seq kit v2 (Life Technologies), using 500 ng of rRNA-subtracted products, as suggested by the manufacturer. Sequences were obtained in the Ion Proton™ platform at Genoinseq (Cantanhede, Portugal).

### Normalization of the data from mRNA libraries

Ion Proton adapter sequences and low-quality bases were trimmed using the Torrent Suite software (Life Technologies). Trimmed reads were aligned to the strain ON4^T^ genome using TMAP version 4.0.6 (Life Technologies). Reads that mapped to the rRNA regions were excluded. The reads count was accessed using eXpress^86^ and normalized using the Trimmed Mean of M values by edgeR^87^. Transcript expression differences between ON4^T^_MMM_ and ON4^T^_LB_ were considered when |Log2FC|> 2, where Fold Change (FC) is the ratio of normalized expression values of ON4^T^_MMM_ over those of ON4^T^_LB_ for each transcript. Thus, transcripts with Log2FC > 2 are overexpressed in ON4^T^_MMM._, whereas those with Log2FC > −2 are overexpressed in ON4^T^_LB_. The transcripts overexpressed in ON4^T^_MMM_ were mapped to the KEGG database^52^ to identify potential overexpressed metabolic pathways related to ACA degradation.

### Validation of transcriptomic data by RT-qPCR

For validation of libraries data, strain ON4^T^ was grown in MMM and LB. Total RNA was extracted using kit NucleoSpin^®^ RNA (Macherey–Nagel), and the cDNA from each sample was obtained with the Maxima H Minus First Strand cDNA Synthesis Kit, (ThermoScientific) (see Supplementary Material for detailed protocol).

The potential reference genes were selected among those without differences in expression between ON4^T^_MMM_ and ON4^T^_LB_. Three reference genes (*gyr*A, *gyr*B, *rec*A) were selected, and primers were designed using Oligo Explorer (http://www.genelink.com/tools/gl-oe.asp). For each reference gene, the primer pair specificity was assessed in silico, followed by qPCR amplification efficiency checking (Tables S10, S11) using different dilutions of DNA amplicons, and the amplification specificity was assessed using melting curves. The target genes were chosen considering differences in the expression values (|Log2FC|> 2) between ON4^T^_MMM_ and ON4^T^_LB_. The primer choice and design and qPCR amplification efficiency and specificity (Tables S10, S11) for target genes were performed as described for reference genes (see Supplementary Material for detailed protocol).

RT-qPCR was performed in 96-well plates (Thermo Fischer Scientific) on the StepOnePlus system (Applied Biosystems). All RT-qPCR experiments were performed using three biological replicates, two technical replicates, and negative control (without cDNA). For detailed protocol see Supplementary Material.

### Evaluation and validation of reference gene expression stability and fold changes of target genes

The candidate reference genes stability was analysed using RefFinder (https://omictools.com/reffinder-tool), where a ranking of genes stability is obtained considering the data obtained from geNorm, NormFinder, BestKeeper and deltaCt method^88–91^. The relative expression profile of each evaluated target gene was determined and normalized using a geometric mean value obtained from the amplification of the three best reference genes in all the samples (ON4^T^_MMM_ and ON4^T^_LB_, respectively). Relative fold changes in gene expression were calculated using the Pfaffl method^92^, and data are displayed as mean ± standard error (SE).

### Plasmid construction and cloning

All bacterial strains and plasmids used in the conjugants construction are listed in Table S12. The full sequence of the target genes (*chnC* and *hyuA/hyuB*) was amplified using total DNA of strain ON4^T^ as a template and specific primers with a *NdeI* restriction site (Table S13). An A-tailing was added to the purified PCR products and further inserted into pTZ57R/T vector. *Escherichia coli* JM109 was transformed with pTZ57R/T with each insert and recombinants were identified by blue/white colour selection and confirmed by colony PCR (Table S14). Recombinant pTZ57R/T vectors were extracted and further digested with the respective Speedy Restriction Enzymes (NZYTech), according to manufacturer’s instructions (Table S13). The pET-30b(+) vector was restricted according to each target. After ligation, *E. coli* JM109 was transformed with the pET-30b(+) alone or carrying the target genes, and positive clones were selected from LB agar plates containing kanamycin (30 µg/mL) and screened by colony PCR (Table S14). Then, *E. coli* BL21 (DE3) was transformed with pET, pET-*chn*C or pET-*hyu*A/B vectors to further express the targeted genes r. See Supplementary Material for detailed protocol.

### Heterologous expression

Resting cells of *E. coli* BL21(DE3) strain carrying the pET-*chn*C or pET-*hyu*A/B vectors were used to analyse the ability of the enzymes caprolactone hydrolase and hydantoin utilisation protein A/B, encoded by *chnC* and *hyuA/hyuB,* respectively, to degrade caprolactam. The biomass was resuspended (DOλ_600nm_ ≈ 12) in 50 mM of phosphate buffer (pH 7.2) and mixed with 6 mM of caprolactam in 50 mM of phosphate buffer (1:1 ratio). The resting cells were incubated at 30 ºC for 24 h. *E. coli* BL21(DE3) with the pET-30b(+) was used as biotic control. Caprolactam was quantified in cell-free supernatants, collected at 0 and 24 h of incubation, by High Performance Liquid Chromatograph (HPLC, VWR Hitachi Chromaster), following the method previously described^93^. See Supplementary Material for detailed protocol.

## Supplementary Information

Supplementary Information.

## Data Availability

The datasets generated and analysed during the current study are available in the respective databases. The genomes obtained were deposited in GenBank under the accession numbers PXVE00000000 (genome ON4^T^_454_), CP028427 and CP028426 (genome ON4^T^_PacBio_) and PXVD00000000 (genome ON4^(−)^). The IS*Gmo1* sequence was deposited in the ISfinder database (https://www-is.biotoul.fr/). The composite transposon carrying *mol*A was named Tn*6311*, according to the Tn number registry (http://www.ucl.ac.uk/eastman/research/departments/microbial-diseases/tn). The data from the cDNA libraries obtained in this work have been deposited in the NCBI Gene Expression Omnibus (GEO) under accession numbers GSM3307305 and GSM3307306 for ON4^T^_MMM_ and ON4^T^_LB_ data, respectively.
