# Supplementary material for: In silico prediction of the enzymes involved in the degradation of the herbicide molinate by Gulosibacter molinativorax ON4T
Source: Sci Rep. 2022 Sep 15;12:15502. doi: 10.1038/s41598-022-18732-5 (PMC9477822; doi:10.1038/s41598-022-18732-5)
Supplement: Supplementary file 1 — Supplementary Information. [file 41598_2022_18732_MOESM1_ESM.pdf]

In silico **prediction of the enzymes involved in the degradation of the herbicide molinate by** ***Gulosibacter molinativorax*** **ON4^T**

Lopes AR[a,b], Bunin, E[a], Viana AT[a], Froufe H[b], Muñoz-Merida A[c], Pinho D[b], Figueiredo J[a], Barroso C[b,d], Vaz-Moreira, I[e], Bellanger X[f], Egas C[b,d] and Nunes OC[a#]

[a]LEPABE - Laboratory for Process Engineering, Environment, Biotechnology and Energy, Faculty of Engineering, University of Porto, Rua Dr. Roberto Frias, 4200-465 Porto, Portugal

[b]Next Generation Sequencing Unit, Biocant, BiocantPark, Núcleo 04, Lote 8, 3060-197 Cantanhede, Portugal

[c]CIBIO – Research Centre in Biodiversity and Genetic Resources, InBIO, University of Porto, Rua Padre Armando Quintas, nº 7 4485-661 Vairão, Portugal

[d]Center for Neuroscience and Cell Biology, University of Coimbra, 3004-504 Coimbra, Portugal

[e]Universidade Católica Portuguesa, CBQF – Centro de Biotecnologia e Química Fina – Laboratório Associado, Escola Superior de Biotecnologia, Rua Diogo Botelho 1327, 4169-005 Porto, Portugal

[f]Université de Lorraine, CNRS, LCPME, F-54000 Nancy, France

Corresponding author:

Olga C. Nunes

e-mail: opnunes@fe.up.pt

LEPABE – Departamento de Engenharia Química,

Faculdade de Engenharia - Universidade do Porto

4200-465 Porto, Portugal

Telephone: +351 225081917; Fax: +351 225081449

Supplementary Material

Material and Methods

Culture conditions and DNA extraction. For 454 pyrosequencing, strain $ON4^T$ was grown on LAM using the quadrant streak method. After the incubation period (30 °C, 72 h), the biomass (central quadrant) was scrapped from the surface of LAM plates, washed with sterile saline solution (0.85 % NaCl, w/v), centrifuged at 4 ºC, and resuspended in the same solution (~$3.3x10^9$ cells/mL). DNA was extracted using the Illustra bacteria genomicPrep Mini Spin Kit (GE Healthcare), following the manufacturer's protocol for Gram-positive bacteria. Prior to sequencing, the genomic DNA was concentrated by isopropanol precipitation to about 1 µg. The same procedure was used to obtain genomic DNA from strain $ON4^{(-)}$ for Illumina sequencing.

For PacBio sequencing, strain $ON4^T$ was grown in mineral medium B [1] supplemented with 0.2 g/L yeast extract and 1.5 mM molinate (MMM). Briefly, four 250 mL Erlenmeyer flasks containing 30 mL of MMM were inoculated with a suspension of strain $ON4^T$ cells previously grown in LAM to reach an initial optical density (λ_{610nm}) of ~0.08±0.01. After incubation at 30 °C, 120 rpm, 24 h, the exponentially growing biomass was pooled together by centrifugation and aliquoted (~3.3 $x10^9$ cells, each). DNA was extracted from four aliquots, using the GRS Genomic DNA Kit - Bacteria (GRiSP), following the manufacturer's protocol for Gram-positive bacteria, except for lysis, which was performed with a final concentration of 10 mg/mL of lysozyme. The genomic DNA from the four aliquots was pooled together in the elution step.

Confirmation of the *mol*A gene regulatory region. Strain ON4^T was grown in MMM, and RNA was extracted from ~1x10^9 exponential growing cells using the RNeasy Mini Kit (Qiagen), following the manufacturer's protocol for "Enzymatic lysis and proteinase K Digestion of Bacteria", but extending the incubation period with 20 mg/mL lysozyme to 1 h. The 5' RACE (5′-Rapid Amplification of cDNA Ends) was performed using the FirstChoice® RLM-RACE kit (Thermo Fisher Scientific), following the small-scale reaction (~1 µg RNA) protocol with few modifications. Briefly, following CIP (calf intestine alkaline phosphatase) reaction, RNA was extracted with 1:1 phenol:chloroform. In addition, 2 µL of target DNA were used in each PCR reaction instead of the described 1 µL. The gene-specific primers used in the outer and inner 5' RLM-RACE PCR nested reactions were R498 (5′-ACTCATGACGGTACGCGCCTGCAT-3′) and R301 (5'-GGCTTCCTCGATGACCTTG-3'), respectively. The inner 5' RLM-RACE PCR reaction product was visualized on a 2% agarose gel stained with ethidium bromide. The obtained amplicon (300-400 bp) was purified and sequenced (ABI 3700 DNA Analyser, Applied Biosystems, CA, USA) using the primer R301.

Phylogenetic analysis of the transposase of IS*Gmo1*. For reconstruction of the transposase phylogenetic tree, a BLASTp search of the predicted amino acid sequence of ORFA, ORFB, and ORFAB protein of IS*Gmo1* was performed in ISFinder (https://www-is.biotoul.fr/). The first 40 closest amino acid sequences of those predicted ORFA, ORFB, and ORFAB proteins were used to build each phylogenetic tree. The amino acid sequences were aligned using MEGA7 [2] with clustalW [3]. Phylogenetic relationships were inferred by the Maximum Likelihood (ML) Phylogeny Test using MEGA7 [2]. Prior to ML analysis, the best protein substitution model of Jones-Taylor-Thornton (JTT) was selected. Branch support was determined by 100 bootstrap replications.

Evaluation of molinate degrading activity of strains *Gulosibacter molinativorax* ON4^T and ON4$^{(-)}$. *G. molinativorax* strains ON4^T and ON4$^{(-)}$ were recovered in Luria-Bertani Agar supplemented with 1 mM molinate (LAM) from 15% glycerol suspensions preserved at -80 ºC. The cultures were incubated at 30 ºC for 72 h, and their purity was confirmed using the Gram-staining technique.

The ability of axenic cultures of strains ON4^T and ON4$^{(-)}$ to degrade molinate was evaluated in 100 mL Erlenmeyer flasks, in duplicate for each tested media. Each Erlenmeyer flask contained 10 mL of mineral medium B [1] supplemented with 1.0 mM molinate and 0.2 g/L yeast extract (MMM) or 10 mL of Luria-Bertani Broth supplemented with 1 mM molinate (LBM). The cell suspensions used as inocula were prepared as described above. Non-inoculated MMM and LBM were used as abiotic controls. All the cultures were incubated at 30 ºC, 120 rpm. Samples were collected at 0, 24, 48, and 72 h of incubation to determine molinate content [1] and the bacterial growth ($OD_{610\ nm}$) over time.

Growth conditions, RNA extraction and sequencing of cDNA libraries. Strain ON4^T was grown at 30 °C, 120 rpm, up to the exponential phase in MMM ($ON4^T_{MMM}$) and Luria-Bertani Broth ($ON4^T_{LB}$) in 250 mL Erlenmeyer flasks to obtain mRNA libraries. About 15-20 mL of culture were centrifuged at 9000 rpm, for 10 min, at 4 ºC, and the resulting cell pellet was stabilized with RNAprotect® Cell Reagent (Qiagen, with a proportion of 1:1). Aliquots of this mixture (1.5 mL) were frozen with liquid N_2 and preserved at -80 ºC until RNA extraction. Total RNA was extracted using the RNeasy extraction kit (QIAGEN GmbH), according to the manufacturer's instructions. Column purification and on-column DNAse I (Life Technologies) digestion was carried out to

remove any impurities from the RNA. Extracted RNA was quantified in Qubit Fluorometer (Invitrogen) with the Qubit® RNA HS assay kit (Invitrogen).

mRNA enrichment by subtractive hybridization

DNA extraction. Genomic DNA was extracted from ~1x10^9 mid-exponential cells grown in MMM using Qiagen DNeasy Blood and Tissue Kit (QIAGEN GmbH, Hilden, Germany) as described in the manufacturer's instructions, with some modifications to improve cell lysis (20 µL of proteinase K were added and the suspension was incubated at 65 ºC, 1000 rpm, for 1.5 h). Genomic DNA was quantified as previously described and further used as a template for the amplification of the 16S and 23S rRNA genes.

Amplification of 16S and 23S rRNA genes to obtain rRNA probes for subtractive hybridization. The probe synthesis reaction was performed as described by Stewart et al. [4]. Briefly, templates for probe generation were first prepared by PCR using primers (Table S15) flanking nearly the full length of the 16S rRNA gene and ~85% of the 23S rRNA gene, with reverse primers modified to contain the T7 RNA polymerase promoter sequence (Table S15). PCRs mixtures (50 µL each) contained 5 µL of 10x AdvantageBufferSA, 3 µL of DMSO, 2 µL of dNTPs mix 5 mM, 2 µL of each primer (27F/1525HS or 256F/2490HS, Table S15) 5 µM, 1 µL of 50x Advantage2 Polymerase Mix, 32.5 µL of ultrapure water and 2.5 µL of template DNA (~50 ng). Reaction conditions were as follows: 5 min at 95 ºC, followed by 30 cycles of 1 min at 94 ºC, 30 s at 57 ºC and 1.5 min (16S rRNA) or 2 min (23S rRNA) at 68 ºC, and a final extension step of 10 min at 68 ºC. The resulting products were visualized *via* gel electrophoresis and purified *via* the Agencourt AMPure XP purification kit (Beckman Coulter) with a proportion of 1:1 v/v (sample:AMPure beads), as described by the manufacturer´s. The

quantity of the 16S and 23S rRNA probes was obtained as previously described (76.2 and 82.4 ng/µL, respectively).

Biotinylated antisense rRNA probes were generated by *in vitro* transcription with T7 RNA polymerase using T7 promoter-containing 16S and 23S rRNA amplicons as templates, using the MEGAscript High Yield Transcription kit (Ambion), as described elsewhere [4] with the following modifications. Briefly, probes for 16S and 23S rRNA genes were generated separately in 20 µL reactions each containing: 1x buffer, T7 RNA polymerase, SUPERase•In™ RNase inhibitor (10 U), ATP (7.5 mM), GTP (7.5 mM), CTP (5.625 mM), UTP (5.625mM), biotin-11-CTP (1.875 mM, Roche), biotin-16-UTP (1.875 mM, Roche) and 16S/23S rDNA template (250–500 ng). Reactions were run at 37 ºC for 4–5 h, then DNA digested with TURBO DNAse (Ambion) for 15 min at 37 ºC. Products were purified using the MEGAclear kit (Ambion), and the elution buffer was pre-heated at 95 ºC (to increase the purification yield). For each probe, two independent elutions were performed with 50 and 30 µL of elution buffer, respectively. The products were quantified by Nanodrop and were kept at -80 ºC until further use.

Subtractive hybridization. Biotinylated rRNA probes were hybridized to complementary rRNA molecules in the total RNA samples (MMM and LB), as described by Stewart et al. [4]. Nevertheless, a second subtractive hybridization on the non-rRNA was established and performed similarly to enrich mRNA. Finally, the obtained purified product was analysed in the Bioanalyzer (Agilent, Santa Clara, CA, USA) to evaluate subtraction efficiency and was kept at -80 ºC until further use in the cDNA synthesis.

Extraction of RNA for validation of transcriptomic data by RT-qPCR. Total RNA was extracted using kit NucleoSpin® RNA (Macherey-Nagel), following the

manufacturer protocol, with some exceptions. RNA purity and integrity were assessed by gel electrophoresis (2% agarose) using 200 ng of total RNA for each sample. Further, the cDNA from each sample was obtained from 1.2 µg of RNA, using Maxima H Minus First Strand cDNA Synthesis Kit (ThermoScientific) with random primers according to the manufacturer's instructions. Three reverse transcription reactions were run simultaneously and were pooled together for each sample. Quantification was performed using the Qubit® RNA HS assay kit (Invitrogen). Simultaneously for each sample, a NRT control reaction was prepared.

Primer design for validation of transcriptomic data by RT-qPCR. Two sets of primers were designed for each reference (gene Oligo explorer; http://www.genelink.com/tools/gl-oe.asp). The first set of primers generated large amplicons between 300-3000 bp, which were further used as standard templates for qPCR of these genes (Table S10). The second set of primers was specifically designed for qPCR assays amplifying small fragments (~100 bp region) (Table S11). The specificity of the primer pairs was assessed *in silico*, using the BLAST PRIMER tool of NCBI (https://www.ncbi.nlm.nih.gov/tools/primer-blast/). Then, qPCR amplification efficiency (Table S11) was checked using different dilutions of DNA amplicons (of each reference gene) and melting curves were obtained to check amplification specificity.

RT-qPCR conditions for validation of transcriptomic data. The reaction mixture was prepared using 10 µL of NZYSpeed qPCR Green Master Mix (2x) ROX plus (NZYtech), 0.4 µL of each primer (10 µM), 2 µL of cDNA (0.28 ng), 0.1 µL of BSA (20 mg/mL) and water to complete a final volume of 20 µL. Cycling conditions were 95 ºC for 3 min, and

40 cycles of 95 ºC for 5 sec, 60 ºC for 30 sec. Primer sequences and characteristics are shown in supplementary Tables S10 and S11.

Determination of absolute plasmid copy number, *mol*A gene per plasmid and 16S rRNA per chromosome in strain ON4^T. To estimate the plasmid copy number and validate the predicted 3 copies of 16S rRNA and *mol*A based on *in silico* analysis, strains ON4^T and ON4$^{(-)}$ were grown in (LAM) and DNA was extracted, as previously described (GRS Genomic DNA Kit – Bacteria, GRiSP). Four genes were selected: *molA* and *parA* (plasmid genes, contig B) and 16S rRNA and *recA* (chromosomic genes, contig A). Given that both *par*A and *rec*A are single-copy genes in each replicon, thus, a ratio of 3 was expected for *molA*:*parA* and 16S rRNA:*recA*, respectively. The primers used and the qPCR amplification efficiencies per gene are described in Tables S10 and S11. In general, the same conditions described for transcriptome validation, except the use of DNA instead of cDNA, were used. The experiments were performed using two biological replicates, and two technical replicates with two different dilutions and an absolute quantification of each gene was performed instead of relative quantification.

Plasmid construction. The full sequence of the target genes (*chnC* and *hyuA/hyuB*) was amplified using total DNA of *G. molinativorax* ON4^T as a template, using specific primers with a *NdeI* restriction site (Table S13), which allowed insertion into the start codon (ATG) of pET-30b(+). The PCR master mix (50 µL) contained: 5 µL of 10x reaction buffer, 2.5 µL of $MgCl_2$ (50 mM), 1.5 µL of dNTPs (10 mM), 1 µL (*chnC*) or 1.3 µL (*hyuA/hyuB*) of each primer (10 µM), 1.25 µL of dimethyl sulfoxide (DMSO), 0.5 µL (*chnC*) or 0.75 µL (*hyuA/hyuB*) of NZYSpeedy Proof DNA Polymerase, 1.22 ng (*chnC*) or 6.1 ng (*hyuA/hyuB*) of template DNA and water nuclease-free up to 50 µL. The

parameters of amplification were: initial denaturation at 95 ºC for 2 min, followed by 30 cycles of denaturation at 95 ºC for 40 sec, annealing at 70 ºC (c*hnC*) or 68 ºC (*hyuA/hyuB*) for 30 s, polymerization at 70 ºC for 15 s (*chnC*) or 65 s (*hyuA/hyuB*) and a final extension step at 70 ºC for 10 min. PCR products were resolved on 1.5% agarose gels and fragments were excised and purified, according to the manufacturer's instructions (GRS PCR & Gel Band Purification Kit, Grisp).

An A-tailing was added to the purified PCR products using the following reaction mixture: 5 µL of the purified DNA fragment, 1 µL of 10x PCR buffer (Mg^{2+}-free), 1 µL of $MgCl_2$ (50 mM), 1 µL of dATPs (2 mM), 0.1 µL of Taq polymerase (5 U/µL) and water nuclease-free up to 10 µL. The mixture was incubated at 72 ºC for 25 min, purified and inserted into pTZ57R/T vector using Thermo Scientific InsTAclone PCR Cloning Kit according to the manufacturer's instructions. The ligation step was modified to 16 h at 14 ºC.

Competent cells of *Escherichia coli* JM109 were transformed with pTZ57R/T with each insert by heat shock using 50 µL of cells and 5 µL of ligation product or 50 ng of pure plasmid, incubated for 30 min on ice, 90 s at 42 ºC and 2 min on ice. After heat shock, 900 µL of SOC medium was inoculated with competent cells and incubated at 37 ºC, 120 rpm, for 1 h. This mixture was plated on LB agar supplemented with ampicillin (50 mg/mL), X-Gal (20 mg/mL) and isopropyl-β-D-thio-galactoside (IPTG) (100 mM). Successful recombinants were identified by blue/white colony color selection. Positive clones were confirmed by colony PCR using NZYTaq II DNA Polymerase with 10x Reaction Buffer (NZYtech) and primers M13F-pUC and M13R-pUC (Table S13). The PCR master mix (50 µL) contained: 5 µL of 10× Reaction Buffer, 4 µL of $MgCl_2$ (50 mM), 1.5 µL of dNTPs (10 mM), 2 µL of each primer (10 µM), 1.25 µL of DMSO, 1.5 µL of NZYTaq II DNA polymerase (1U/µL) and water nuclease-free up to 50 µL. The

parameters of amplification were: initial denaturation step at 94 ºC for 2 min, followed by 30 cycles of denaturation at 94 ºC for 40 s, annealing at 56 ºC for 30 s, and polymerization at 72 ºC for 1 min (*chnC*) or 3 min (*hyuA/hyuB*), and a final extension step at 72 ºC for 10 min.

One of the positive clones was used to extract the target plasmid, which was further digested with the respective Speedy Restriction Enzymes (NZYTech), according to the manufacturer's instructions (Table S14). The pET-30b(+) vector was restricted according to each target. In the digestion reaction, 1 µg of DNA was used. The digested products were subjected to electrophoresis, excised, and purified. The insert and plasmid similarly digested were ligated in a 3:1 molar ratio proportion with T4 DNA Ligase (Thermo Scientific) according to the manufacturer's instructions, at 14 ºC for 16 h. *E. coli* JM109 was transformed with 5 µL of ligation product or 50 ng of pure plasmid by heat shock, as described above. Clones were selected from LB agar plates containing kanamycin (30 µg/mL) after overnight growth at 37 ºC and confirmed by colony PCR using NZYTaq II DNA Polymerase with (NZYtech) and primers T7 promoter and T7 terminator (Table S14). The PCR master mix and the parameters of amplification were the same used in the first colony PCR. One of the positive clones was used for plasmid extraction and was further used to transform *E. coli* BL21 (DE3). Colony PCR was performed to choose one clone for further studies. *E. coli* BL21(DE3) strain carrying either pET-30b(+), pET-*chn*C or pET-*hyu*A/B vectors were stored at -80 °C in 15% (v/v) glycerol.

Preparation of the transformants for the resting cell assays. Stored *E. coli* BL21(DE3) strain carrying either pET-30b(+), pET-*chn*C or pET-*hyu*A/B vectors were plated in Luria Bertani (LB) Agar supplemented with kanamycin (30 µg/mL) and incubated overnight, at 37 ºC. Pre-cultures of each transformant were obtained by single colony inoculation,

in LB with kanamycin, incubated overnight, at 37 ºC and 120 rpm. Then, 1 mL of each pre-culture was used to inoculate 50 mL of LB with kanamycin, incubated at 37 ºC until reaching an optical density ($DO\lambda_{600nm}$) between 0.5 and 0.7 ± 0.01. At this point, cultures were induced using IPTG (1 mM) and incubated at 30 ºC, for 20 h at 120 rpm. Then, each culture was centrifuged at 12 000 rpm for 10 min at 8 ºC and the pellets were further used in the resting cell assays.

Table S1. Metrics from genome sequence assemblies used in the present study. Completeness and contamination metrics were obtained with CheckM.

Genome	Sequencing technology	Genome size (bp)	Longest contig (bp)	No. of contigs	Total predicted genes	Completeness (%)	Contamination (%)	Accession number
$ON4^{T}_{454}$	454 pyrosequencing	3,427,741	170,114	163	3246	98.64	1.17	Present study (PXVE00000000)
$ON4^{T}_{PacBio}$	PacBio	3,502,671	3,465,658	2	3301	98.64	0.58	Present study (CP028426 and CP028427)
$DSM13485^{T}$ ($=ON4^{T}$)	Illumina	3,378,766	210,143	82	3169	98.64	0.58	(AUDX00000000.1)
$ON4^{(-)}$	Illumina	3,405,077	210,102	79	3174	98.64	0.58	Present study (PXVD00000000)

Table S2. Estimated number of 16S rRNA gene copies in the chromosome of strains ON4^{T} and ON4$^{(-)}$ determined by the ratio between 16S rRNA:*recA*. Estimated copy number of *mol*A given by the ratios between *molA*:*parA* and of plasmid per cell given by the ratio between *parA*:*recA*, in the strain ON4^{T}, obtained by qPCR quantification of 16S rRNA, *rec*A, *mol*A, and *par*A genes.

Gulosibacter molinativorax **ON4$^{(-)}$**	***Gulosibacter molinativorax*** **ON4^{T}**		
**16S rRNA:*recA***	**16S rRNA:*recA***	***molA*:*parA***	***parA*:*recA***
3.1 ± 0.11	2.9 ± 0.21	2.8 ± 0.14	2.7 ± 0.31

Table S3. Similarity between strain ON4^T genome (contig A+B, contig A or contig B) with that of *A. casei* LMG 22410^T draft genome (FUHU00000000.1) and contig 18 (FUHU01000018.1) based on average nucleotide identity (ANI) values

	OrthoANIu value (%)*	Average aligned length (bp)	Genome coverage (%)
	Agrococcus casei LMG 22410^T (FUHU00000000.1)		
Strain ON4^T contig A+contig B	71.5	433,584	15.7
Strain ON4^T contig A	70.8	416,584	15.1
Strain ON4^T contig B	88.2	17,000	0.61
	A. casei LMG 22410^T (FUHU00000000.1, Contig FUHU01000018.1)		
Strain ON4^T contig B	88.2	17,000	53.8

* The values were obtained using ANI calculation (https://www.ezbiocloud.net/tools/ani)[5]

Table S4. Genetic organization of pARLON1. Grey shadowing indicate open reading frames (*orf*) with homology with those of contig FUHU01000018.1 from *A. casei* LMG 22410^T (FUHU00000000.1) draft genome. Blue letters indicate *orf* related to a putative T4SS of the type$_{FATA}$, as predicted by CONJscan [6] and/or oriTDB [7].

Locus Tag	Product	ORF	ORF from contig18 *A. casei* with homology with those from contig B	Predicted ORFs by CONJscan and/or oriTDB
GMOLON4_3255	Permease	*orf*1	*orf*9	FATA_prgF
GMOLON4_3256	Hypothetical protein	*orf*2	*orf*8	homology with VirB6 (T4SS)
GMOLON4_3257	Prgl family	*orf*3	-	homology with VirB3 (T4SS)
GMOLON4_3258	Transfer complex protein	*orf*4	*orf*7	VirB4 ATPase (T4SS)
GMOLON4_3259	M23/M37 family peptidase	nlpD	*orf*6	-
GMOLON4_3260	Hypothetical protein	orf6	*orf*5	-
GMOLON4_3261	ATPase; FHA family	orf7	*orf*4	-
GMOLON4_3262	ATPase	*orf*8	*orf* 3	-
GMOLON4_3263	Hypothetical protein	*orf*9	*orf* 2	-
GMOLON4_3264	MaR family transcriptional regulator	*orf*10	*orf* 1	-
GMOLON4_3265	Hypothetical protein	*orf*11	*orf*39	-
GMOLON4_3266	Hypothetical protein	*orf*12	*orf*38	-
GMOLON4_3267	Hypothetical protein	*orf*13	*orf*37	-
GMOLON4_3268	Hypothetical protein	*orf*14	*orf* 36	-
GMOLON4_3269	Hypothetical protein	*orf*15	*orf*35	-
GMOLON4_3270	Hypothetical protein	*orf*16	*orf*34	-
GMOLON4_3271	Hypothetical protein	*orf*17	*orf*33	-
GMOLON4_3272	Hypothetical protein	*nrd*H	*orf* 32	-
GMOLON4_3273	Hypothetical protein	*orf*19	*orf*31	-
GMOLON4_3274	Nuclease (SNase domain-containing protein)	*orf*20	*orf* 30	-
GMOLON4_3275	Single-stranded DNA-binding protein	*orf*21	*orf* 29	-
GMOLON4_3276	Plasmid partition protein homolog ParA	*par*A	*orf* 28	-
GMOLON4_3277	Hypothetical protein	*orf*23	*orf*27	-
GMOLON4_3278	Hypothetical protein	*orf*24	*orf* 26	-
GMOLON4_3279	type II toxin-antitoxin interferase; sodium-indep. anion	*orf*25	-	-
GMOLON4_0010	Transposase IS3/IS911 family protein	*orf*26	-	-
GMOLON4_0005	Transposase	*orf*27	-	-
GMOLON4_3280	Molinate hydrolase	*mol*A	-	-
GMOLON4_0020	Transposase IS3/IS911 family protein	*orf*29	-	-
GMOLON4_0015	Transposase	*orf*30	-	-
GMOLON4_3281	Molinate hydrolase	*mol*A	-	-
GMOLON4_0030	Transposase IS3/IS911 family protein	*orf*32	-	-
GMOLON4_0025	Transposase	*orf*33	-	-
GMOLON4_3282	Molinate hydrolase	*mol*A	-	-
GMOLON4_0040	Transposase IS3/IS911	*orf*35	-	-
GMOLON4_0035	Transposase	*orf*36	-	-
GMOLON4_3283	Phospholipid carrier-dependent; glycosyltranferase	*orf*37	-	-
GMOLON4_3284	alpha manosidase	*orf*38	*orf*17	-

GMOLON4_3285	Adenosine monophosphate-protein transferase VbhT	*fic*	*orf* 16	-
GMOLON4_3286	Resolvase	*orf*40	*orf*15	-
GMOLON4_3287	Hypothetical protein	*orf*41	*orf*14	-
GMOLON4_3288	Hypothetical protein	*orf*42	-	-
GMOLON4_3289	plasmid mobilization; relaxosome	*mob*C	*orf*13	-
GMOLON4_3290	relaxase	*rlx*	*orf* 12	homology with MOB
GMOLON4_3291	DUF3801; PcfB family	orf45	*orf1*1	FATA_cd411
GMOLON4_3292	TraG/TraD family	*trs*K	*orf*10	TraG/TraD family protein (T4CP)
GMOLON4_3293	Hypothetical protein	*orf*47	-	-

Table S5. Metrics of the genes expressed in strain $ON4^T$ grown in the presence and absence of molinate.

	Number of genes	% of genes*
Total expressed protein-coding genes genes ($ON4^T_{MMM}$ and/or $ON4^T_{LB}$)	3095	95.4
Protein-coding genes expressed in both $ON4^T_{MMM}$ and $ON4^T_{LB}$	2726	84.1
Protein-coding genes not expressed	148	4.6
Protein-coding genes with similar expression in $ON4^T_{MMM}$ and $ON4^T_{LB}$	2398	73.9
Protein-coding genes overexpressed in $ON4^T_{MMM}$ *vs* $ON4^T_{LB}$	335	10.3
Chromosomal genes only expressed in $ON4^T_{MMM}$	121	3.7
Plasmid genes only expressed in $ON4^T_{MMM}$	28	0.9
Protein-coding genes overexpressed in $ON4^T_{LB}$ *vs* $ON4^T_{MMM}$	362	11.2
Chromosomal genes only expressed in $ON4^T_{LB}$	220	6.8
Plasmid genes only expressed in $ON4^T_{LB}$	0	0

* Based on the strain $ON4^T$ genome ($ON4^T_{PacBio}$ – CP028426 and CP028427)

Table S6. Chromosomal genes overexpressed in strain ON4^T growing in MMM over those growing in LB.

Locus Tag	Gene	Product	LOG2FC#
GMOLON4_31	GMOLON4_31	Transglycosylase domain-containing protein	2.52
GMOLON4_57	*erm*A	Dimethyladenosine transferase (RRNA methylation)	ND
GMOLON4_64	GMOLON4_64	Possibl zinc metallo-peptidase	2.41
GMOLON4_77	*yxj*L	Uncharacterized transcriptional regulatory protein yxjL	2.19
GMOLON4_79	GMOLON4_79	Putative PadR family transcriptional regulator	2.19
GMOLON4_92	GMOLON4_92	Hypothetical protein	ND
GMOLON4_118	GMOLON4_118	Hypothetical protein	2.16
GMOLON4_121	GMOLON4_121	Hypothetical protein	ND
GMOLON4_122	GMOLON4_122	Hypothetical protein	ND
GMOLON4_123	GMOLON4_123	ParB-like protein	4.44
GMOLON4_124	GMOLON4_124	Hypothetical protein	ND
GMOLON4_125	GMOLON4_125	Hypothetical protein	ND
GMOLON4_126	GMOLON4_126	Hypothetical protein	ND
GMOLON4_128	GMOLON4_128	Hypothetical protein	2.51
GMOLON4_132	GMOLON4_132	Hypothetical protein	3.00
GMOLON4_134	GMOLON4_134	Hypothetical protein	2.36
GMOLON4_136	GMOLON4_136	Hypothetical protein	ND
GMOLON4_137	GMOLON4_137	Hypothetical protein	ND
GMOLON4_139	GMOLON4_139	Hypothetical protein	ND
GMOLON4_140	GMOLON4_140	DnaB domain protein helicase domain protein	2.51
GMOLON4_141	GMOLON4_141	Hypothetical protein	ND
GMOLON4_142	GMOLON4_142	Hypothetical protein	ND
GMOLON4_146	GMOLON4_146	Hypothetical protein	ND
GMOLON4_151	GMOLON4_151	Hypothetical protein	ND
GMOLON4_155	GMOLON4_155	Hypothetical protein	ND
GMOLON4_157	GMOLON4_157	Hypothetical protein	ND
GMOLON4_158	GMOLON4_158	Hypothetical protein	ND
GMOLON4_159	GMOLON4_159	Hypothetical protein	2.19
GMOLON4_165	GMOLON4_165	Hypothetical protein	ND
GMOLON4_167	GMOLON4_167	Hypothetical protein	ND
GMOLON4_168	GMOLON4_168	Hypothetical protein	8.51
GMOLON4_170	GMOLON4_170	Hypothetical protein	ND
GMOLON4_179	GMOLON4_179	Hypothetical protein	3.73
GMOLON4_187	*ogt*	Methylated-DNA--protein-cysteine methyltransferase	2.19
GMOLON4_204	*yfc*H	NAD dependent epimerase/dehydratase family protein	2.30
GMOLON4_205	*des*A3	Stearoyl-CoA 9-desaturase	2.19
GMOLON4_230	*gsi*A	ABC transporter ATP-binding protein	2.77
GMOLON4_231	*gsi*D	Glutathione transport system permease protein GsiD	2.72
GMOLON4_233	*opp*A	Peptide/nickel transport system substrate-binding protein	2.56
GMOLON4_237	*eth*R	HTH-type transcriptional regulator EthR	2.89
GMOLON4_277	GMOLON4_277	Hypothetical protein	2.06
GMOLON4_305	GMOLON4_305	Hypothetical protein	ND
GMOLON4_307	*nud*L	Putative Nudix hydrolase NudL	2.08
GMOLON4_365	GMOLON4_365	Amino acid permease	2.32

Table S6. Continue

Locus Tag	Gene	Product	LOG2FC#
GMOLON4_378	GMOLON4_378	Hypothetical protein	2.36
GMOLON4_402	GMOLON4_402	Hypothetical protein	2.77
GMOLON4_417	GMOLON4_417	Hypothetical protein	ND
GMOLON4_428	GMOLON4_428	Hypothetical protein	ND
GMOLON4_430	*kdp*D	Histidine kinase	4.51
GMOLON4_431	*agu*A	Agmatine deiminase	4.75
GMOLON4_432	GMOLON4_432	Hypothetical protein	ND
GMOLON4_433	*puu*P	Amino acid permease-associated region	7.50
GMOLON4_434	GMOLON4_434	Glutamine amidotransferase	6.84
GMOLON4_435	*cin**	Nitric oxide synthase	5.85
GMOLON4_436	*fpr*A*	NADPH-ferredoxin reductase FprA	7.44
GMOLON4_437	*bio*I	Cytochrome P450	6.10
GMOLON4_438	*gln*A_2	Glutamine synthetase	6.29
GMOLON4_439	GMOLON4_439	Hypothetical protein	7.06
GMOLON4_440	GMOLON4_440	Peptidase C26	6.74
GMOLON4_441	*gua*A	Glutamine amidotransferase class I	5.39
GMOLON4_444	*kgd*	Alpha-ketoglutarate decarboxylase	ND
GMOLON4_447	GMOLON4_447	Integrase family protein	2.77
GMOLON4_448	GMOLON4_448	Cro/Cl family transcriptional regulator	ND
GMOLON4_449	GMOLON4_449	Hypothetical protein	ND
GMOLON4_451	*ars*X	Thioredoxin	ND
GMOLON4_452	*ars*B	Arsenite export protein	4.00
GMOLON4_453	*ars*R	Putative arsenic resistance operon repressor ArsR	ND
GMOLON4_454	*chr*A	Chromate transport protein ChrA	ND
GMOLON4_456	*ars*C	Low molecular weight phosphotyrosine protein phosphatase	ND
GMOLON4_457	*dau*A	C4-dicarboxylic acid transporter DauA	ND
GMOLON4_458	*znt*R	HTH-type transcriptional regulator ZntR	ND
GMOLON4_464	GMOLON4_464	Hypothetical protein	3.10
GMOLON4_471	GMOLON4_471	Excisionase	ND
GMOLON4_477	*pro*S_1	Aminoacyl-tRNA deacylase	3.89
GMOLON4_479	GMOLON4_479	Helix-turn-helix family protein	2.19
GMOLON4_484	GMOLON4_484	Histidine kinase	2.28
GMOLON4_490	*xer*D_2	Tyrosine recombinase XerD	ND
GMOLON4_505	GMOLON4_505	Asparagine synthetase [glutamine-hydrolyzing] AsnH	ND
GMOLON4_506	GMOLON4_506	ABC transporter substrate-binding protein	ND
GMOLON4_508	*ytr*E	ABC transporter ATP-binding protein YtrE	ND
GMOLON4_510	*adh*A	Putative alcohol dehydrogenase AdhA	ND
GMOLON4_531	*ubi*E	UbiE/COQ5 family methlytransferase	ND
GMOLON4_542	*amt*B	Ammonium transporter	ND
GMOLON4_545	*sis*32	ArsR family transcriptional regulator	2.51
GMOLON4_549	*ubi*G	3-demethylubiquinone-9 3-O-methyltransferase	2.51
GMOLON4_552	GMOLON4_552	Transcriptional regulator, LysR family	2.06
GMOLON4_575	*fad*D	Long-chain acyl-CoA synthetase	2.13
GMOLON4_651	*ybb*K	Putative activity regulator of membrane protease YbbK	ND

Table S6. Continue

Locus Tag	Gene	Product	LOG2FC#
GMOLON4_653	*fad*B	3-hydroxyacyl-CoA dehydrogenase	2.05
GMOLON4_680	*lig*T	LigT	ND
GMOLON4_681	GMOLON4_681	Hypothetical protein	2.51
GMOLON4_694	GMOLON4_694	Hypothetical protein	ND
GMOLON4_744	*thc*A	Non-phosphorylating glyceraldehyde-3-phosphate dehydrogenase (NADP)	2.37
GMOLON4_774	*cbi*Q	ABC-type cobalt transport system, permease protein	2.75
GMOLON4_775	*bio*M	Biotin transport ATP-binding protein BioM	2.41
GMOLON4_776	*bio*Y	Uncharacterized conserved protein	3.16
GMOLON4_777	*tet*R	Transcriptional regulator	2.22
GMOLON4_802	*tra*I	Multifunctional conjugation protein TraI	ND
GMOLON4_804	GMOLON4_804	Hypothetical protein	ND
GMOLON4_805	GMOLON4_805	Hypothetical protein	ND
GMOLON4_816	*ins*F	Transposase InsF for insertion sequence IS3	6.72
GMOLON4_817	GMOLON4_817	Transposase IS3/IS911	ND
GMOLON4_884	*nfd*A_2	Amidohydrolase	3.84
GMOLON4_885	*paa*J	3-oxoadipyl-CoA/3-oxo-5,6-dehydrosuberyl-CoA thiolase	4.18
GMOLON4_886	*mmg*C	Acyl-CoA dehydrogenase MmgC	4.94
GMOLON4_887	*bbs*F	Succinyl-CoA:(R)-benzylsuccinate CoA-transferase subunit BbsF	4.21
GMOLON4_889	*pla*P	Low-affinity putrescine importer PlaP	3.33
GMOLON4_890	*adh*	S-(hydroxymethyl)glutathione dehydrogenase	2.26
GMOLON4_893	GMOLON4_893	PucR family transcriptional regulator	2.98
GMOLON4_894	GMOLON4_894	Predicted TIM-barrel fold metal-dependent hydrolase	4.69
GMOLON4_895	*yhj*E	MFS transporter	4.59
GMOLON4_900	*atr*B	Putative amino acid ABC transporter permease protein	ND
GMOLON4_903	GMOLON4_903	Auxin efflux carrier	5.65
GMOLON4_904	*aru*I	Acetolactate synthase	5.97
GMOLON4_905	*ser*A	Dehydrogenase	6.44
GMOLON4_906	*fbp*C	Fe(3+) ions import ATP-binding protein FbpC	7.96
GMOLON4_907	*pot*C	Spermidine/putrescine transport system permease protein potC	7.61
GMOLON4_908	*pot*B	ABC transporter permease	3.93
GMOLON4_909	GMOLON4_909	Polyamine ABC transporter substrate-binding protein	7.99
GMOLON4_910	*spu*C	Adenosylmethionine-8-amino-7-oxononanoate transaminase	5.89
GMOLON4_911	*mmg*B	Putative 3-hydroxybutyryl-CoA dehydrogenase	ND
GMOLON4_912	*paa*A	Putative enoyl-CoA hydratase PaaA	3.59
GMOLON4_913	*chn*E	6-oxohexanoate dehydrogenase	3.73
GMOLON4_914	*ala*	Ornithine cyclodeaminase	4.25
GMOLON4_915	*sol*A1	Putative sarcosine oxidase	4.96
GMOLON4_916	*puo*	Putrescine oxidase	5.56
GMOLON4_917	*pla*P_2	Large neutral amino acids transporter small subunit 2	6.29
GMOLON4_921	*xyl*B	Putative xylulokinase	2.11
GMOLON4_923	*gat*Y	Fructose-1,6-bisphosphate aldolase	2.60
GMOLON4_926	*rbs*A	Sugar (Ribose) ABC transporter ATP-binding protein	ND
GMOLON4_929	GMOLON4_929	DoxX family protein	ND
GMOLON4_933	GMOLON4_933	Hypothetical protein	ND

Table S6. Continue

Locus Tag	Gene	Product	LOG2FC#
GMOLON4_939	GMOLON4_939	Hypothetical protein	2.19
GMOLON4_953	*fab*G21	3-oxoacyl-[acyl-carrier-protein] reductase	2.62
GMOLON4_973	GMOLON4_973	Hypothetical protein	ND
GMOLON4_990	GMOLON4_990	Conjugal transfer protein TrbL	ND
GMOLON4_1010	GMOLON4_1010	Hypothetical protein	ND
GMOLON4_1038	*cyp*104	Cytochrome P450-pinF2, plant-inducible	3.70
GMOLON4_1052	GMOLON4_1052	Putative membrane protein	2.77
GMOLON4_1068	*lip*A	Lipoyl synthase	5.60
GMOLON4_1069	*lip*B	Octanoyltransferase	3.78
GMOLON4_1106	*mut*Y	A/G-specific adenine glycosylase	2.77
GMOLON4_1156	*czr*B	Cation diffusion facilitator family transporter	2.22
GMOLON4_1157	*hip*B	Helix-turn-helix family protein	2.65
GMOLON4_1158	*met*B	Cystathionine gamma-synthase	2.13
GMOLON4_1182	*yec*C	Amino acid ABC transporter, ATP-binding protein	2.19
GMOLON4_1188	GMOLON4_1188	Hypothetical protein	ND
GMOLON4_1191	GMOLON4_1191	Hypothetical protein	ND
GMOLON4_1219	*ptl*H	Type II secretion system protein E	ND
GMOLON4_1251	*shi*A	Shikimate transporter	ND
GMOLON4_1272	*ahp*F	NADH dehydrogenase	2.05
GMOLON4_1281	*yhd*A	Oxidoreductase, NAD(P)H-FMN and ferric iron reductase	ND
GMOLON4_1324	*nar*J	Nitrate reductase delta subunit	2.65
GMOLON4_1348	GMOLON4_1348	Superfamily I DNA and RNA helicase	3.19
GMOLON4_1351	*bet*I_4	HTH-type transcriptional regulator BetI	ND
GMOLON4_1359	GMOLON4_1359	Hypothetical protein	2.19
GMOLON4_1366	GMOLON4_1366	Antibiotic biosynthesis monooxygenase	ND
GMOLON4_1369	GMOLON4_1369	YhhN-like protein	2.77
GMOLON4_1378	*ohp*A	MFS transporter	2.39
GMOLON4_1380	*vir*F	Virulence regulon transcriptional activator VirF	2.13
GMOLON4_1411	GMOLON4_1411	Hypothetical protein	ND
GMOLON4_1444	GMOLON4_1444	Hypothetical protein	ND
GMOLON4_1457	*paa*H	Putative 3-hydroxybutyryl-CoA dehydrogenase PaaH	2.05
GMOLON4_1492	*chn*B*	Cyclohexanone monooxygenase	6.28
GMOLON4_1493	*chn*C*	Caprolactone hydrolase	3.56
GMOLON4_1494	GMOLON4_1494	Hypothetical protein	5.04
GMOLON4_1495	*bbs*F	Carnitine dehydratase	5.61
GMOLON4_1496	*adh*P*	Alcohol dehydrogenase	5.85
GMOLON4_1497	*sad*	Succinate-semialdehyde dehydrogenase [NAD]	5.75
GMOLON4_1498	*pro*P	Proline porter II	5.04
GMOLON4_1524	GMOLON4_1524	Hypothetical protein	2.51
GMOLON4_1536	GMOLON4_1536	Hypothetical protein	ND
GMOLON4_1539	*met*E*	5-methyltetrahydropteroyltriglutamate-homocysteine methyltransferase	8.80
GMOLON4_1540	GMOLON4_1540	Hypothetical protein	7.55
GMOLON4_1553	GMOLON4_1553	Putative integral membrane protein	ND
GMOLON4_1565	*psp*E	Thiosulfate sulfurtransferase PspE	ND

Table S6. Continue

Locus Tag	Gene	Product	LOG2FC#
GMOLON4_1577	*sig*B	Putative ATP-binding protein	4.10
GMOLON4_1582	*nrd*B	Ribonucleoside-diphosphate reductase subunit beta	ND
GMOLON4_1607	*cad*D	Cadmium resistance protein CadD	2.65
GMOLON4_1632	GMOLON4_1632	DNA-binding protein	ND
GMOLON4_1651	*tat*A	Sec-independent protein translocase protein TatA	ND
GMOLON4_1665	GMOLON4_1665	Helicase, putative	2.06
GMOLON4_1667	GMOLON4_1667	Sll8048 protein	ND
GMOLON4_1673	*tnp*A	Putative transposase	5.88
GMOLON4_1686	GMOLON4_1686	Hypothetical protein	3.00
GMOLON4_1697	GMOLON4_1697	Hypothetical protein	ND
GMOLON4_1797	GMOLON4_1797	Prophage Lp1 protein 5	2.47
GMOLON4_1810	*adh*	Alcohol dehydrogenase	2.52
GMOLON4_1817	*bdp*A	Bacterial regulatory helix-turn-helix s, AraC family protein	2.65
GMOLON4_1826	GMOLON4_1826	Hypothetical protein	4.47
GMOLON4_1827	*dna*Q	Exonuclease, DNA polymerase III, epsilon subunit family	4.04
GMOLON4_1828	*met*Q2	D-methionine-binding lipoprotein MetQ	4.25
GMOLON4_1829	*met*P	Methionine import system permease protein MetP	3.93
GMOLON4_1830	*met*N	DL-methionine transporter ATP-binding subunit	4.89
GMOLON4_1831	GMOLON4_1831	Hypothetical protein	ND
GMOLON4_1832	GMOLON4_1832	Hypothetical protein	ND
GMOLON4_1837	GMOLON4_1837	Toxin-antitoxin system toxin subunit	ND
GMOLON4_1840	GMOLON4_1840	Hypothetical protein	ND
GMOLON4_1848	GMOLON4_1848	Short-chain dehydrogenase reductase 3a	2.14
GMOLON4_1925	GMOLON4_1925	Phosphoribosyltransferase	ND
GMOLON4_1947	GMOLON4_1947	Hypothetical protein	ND
GMOLON4_1949	*yea*O	Uncharacterized protein yeaO	ND
GMOLON4_1954	*mal*F	Maltose transport system permease protein malF	ND
GMOLON4_1984	GMOLON4_1984	Putative regulatory protein	ND
GMOLON4_2001	GMOLON4_2001	Sugar ABC transporter substrate-binding protein	ND
GMOLON4_2020	*adh*C	Alcohol dehydrogenase	2.51
GMOLON4_2088	GMOLON4_2088	Hypothetical protein	ND
GMOLON4_2106	*acd*	Acyl-CoA dehydrogenase	3.49
GMOLON4_2119	GMOLON4_2119	XRE family transcriptional regulator	2.77
GMOLON4_2129	GMOLON4_2129	Hypothetical protein	ND
GMOLON4_2168	GMOLON4_2168	HNH endonuclease	2.65
GMOLON4_2171	*fad*E	Putative acyl-CoA dehydrogenase	3.37
GMOLON4_2228	*mpr*A	Response regulator MprA	ND
GMOLON4_2230	GMOLON4_2230	Hypothetical protein	ND
GMOLON4_2241	*cpn*A	Cyclopentanol dehydrogenase	2.44
GMOLON4_2244	GMOLON4_2244	Hypothetical protein	ND
GMOLON4_2245	GMOLON4_2245	Nucleotidyltransferase domain protein	ND
GMOLON4_2250	*sco*A	3-oxoacid CoA-transferase subunit A	ND
GMOLON4_2254	GMOLON4_2254	Activator of HSP90 ATPase	2.77
GMOLON4_2278	GMOLON4_2278	Putative exported protein	2.19

Table S6. Continue

Locus Tag	Gene	Product	LOG2FC[#]
GMOLON4_2285	*cmp*R	LysR family transcriptional regulator	3.19
GMOLON4_2288	*pcd*	Piperideine-6-carboxylate dehydrogenase	2.13
GMOLON4_2290	GMOLON4_2290	Hypothetical protein	ND
GMOLON4_2293	*yec*S	Amino acid ABC transporter permease protein	2.10
GMOLON4_2349	*bet*I_7	HTH-type transcriptional regulator BetI	ND
GMOLON4_2355	*rpm*J	50S ribosomal protein L36	ND
GMOLON4_2356	GMOLON4_2356	Hypothetical protein	ND
GMOLON4_2359	GMOLON4_2359	HNH endonuclease	ND
GMOLON4_2362	GMOLON4_2362	Hypothetical protein	ND
GMOLON4_2365	*rbs*A	Ribose import ATP-binding protein RbsA	3.42
GMOLON4_2366	*yph*D	Putative ABC transporter permease protein YphD	2.31
GMOLON4_2368	*gdh*B	Quinoprotein glucose dehydrogenase B	2.18
GMOLON4_2369	*phi*	Demethylmenaquinone methyltransferase	3.50
GMOLON4_2370	*rht*B	Lysine Exporter Protein	3.63
GMOLON4_2376	*cnq*744	Deaminase/reductase	3.00
GMOLON4_2496	GMOLON4_2496	Lactoylglutathione lyase	3.65
GMOLON4_2498	*sty*D	Phenylacetaldehyde dehydrogenase	2.67
GMOLON4_2525	*ohp*A	Major facilitator transporter	2.24
GMOLON4_2526	*nfd*A_3	N-substituted formamide deformylase	4.28
GMOLON4_2552	*sox*C	Dibenzothiophene desulfurization enzyme C	2.12
GMOLON4_2554	*sfn*A	Acyl-CoA dehydrogenase/oxidase family protein	2.71
GMOLON4_2560	*rss*A_1	NTE family protein rssA	2.11
GMOLON4_2562	GMOLON4_2562	Thioredoxin	ND
GMOLON4_2588	*pot*H	Binding--dependent transport system inner membrane component family protein	2.77
GMOLON4_2589	*pot*B	Binding--dependent transport system inner membrane component family protein	3.77
GMOLON4_2637	GMOLON4_2637	Hypothetical protein	ND
GMOLON4_2644	GMOLON4_2644	Hypothetical protein	ND
GMOLON4_2646	GMOLON4_2646	Hypothetical protein	ND
GMOLON4_2653	GMOLON4_2653	Hypothetical protein	ND
GMOLON4_2655	GMOLON4_2655	Hypothetical protein	ND
GMOLON4_2656	GMOLON4_2656	Hypothetical protein	ND
GMOLON4_2689	*int*1	Integrase catalytic region	4.19
GMOLON4_2691	GMOLON4_2691	Hypothetical protein	ND
GMOLON4_2693	GMOLON4_2693	ArsR family transcriptional regulator	3.36
GMOLON4_2733	*tat*A	Sec-independent protein translocase protein TatA	ND
GMOLON4_2760	GMOLON4_2760	Hypothetical protein	ND
GMOLON4_2765	*glo*A5	Glyoxalase/bleomycin resistance protein/dioxygenase	4.15
GMOLON4_2768	GMOLON4_2768	DSBA oxidoreductase	ND
GMOLON4_2769	GMOLON4_2769	Hypothetical protein	ND
GMOLON4_2775	GMOLON4_2775	Hypothetical protein	4.09
GMOLON4_2779	*hsa*B	Flavin-dependent monooxygenase, reductase subunit HsaB	ND
GMOLON4_2780	GMOLON4_2780	Hypothetical protein	ND
GMOLON4_2849	GMOLON4_2849	Hypothetical protein	ND
GMOLON4_2892	GMOLON4_2892	Hypothetical protein	ND

Table S6. Continue

Locus Tag	Gene	Product	LOG2FC#
GMOLON4_3003	GMOLON4_3003	Hypothetical protein	2.19
GMOLON4_3011	GMOLON4_3011	Hypothetical protein	ND
GMOLON4_3045	*puu*P	Amino acid permease	2.54
GMOLON4_3046	GMOLON4_3046	Hypothetical protein	2.14
GMOLON4_3048	*rmp*R	DNA-binding regulatory protein RmpR	2.07
GMOLON4_3052	*gln*A_2	Glutamine synthetase	2.19
GMOLON4_3068	*nad*C	Putative nicotinate-nucleotide pyrophosphorylase [carboxylating]	ND
GMOLON4_3084	GMOLON4_3084	Two-component hybrid sensor and regulator	ND
GMOLON4_3085	GMOLON4_3085	Hypothetical protein	2.39
GMOLON4_3086	*tpa*	Taurine--pyruvate aminotransferase	2.84
GMOLON4_3087	*mao*B	Phenylacetaldehyde dehydrogenase	2.77
GMOLON4_3088	*adh*	Alcohol dehydrogenase	2.19
GMOLON4_3089	*dad*A	D-amino acid dehydrogenase small subunit	2.75
GMOLON4_3090	*alr*	Alanine racemase	4.77
GMOLON4_3094	*ipu*C	Gamma-glutamylisopropylamide synthetase	3.51
GMOLON4_3095	*gdh*	Glucose 1-dehydrogenase	ND
GMOLON4_3097	*cpx*P	Cytochrome P450 monooxygenase	3.10
GMOLON4_3098	GMOLON4_3098	Ferredoxin	ND
GMOLON4_3099	GMOLON4_3099	Hypothetical protein	1.60
GMOLON4_3166	GMOLON4_3166	Hypothetical protein	ND
GMOLON4_3195	*tns*	Transposase	ND
GMOLON4_3198	GMOLON4_3198	Hypothetical protein	2.77
GMOLON4_3199	GMOLON4_3199	Acetyltransferase	2.02
GMOLON4_3200	GMOLON4_3200	Protein export cytoplasm protein SecA ATPase RNA helicase	3.19
GMOLON4_3203	*hyu*A*	Hydantoin utilization protein A	3.59
GMOLON4_3204	*hyu*B	Hydantoinase B	2.04
GMOLON4_3205	*hpy*A	Putative permease for 2-hydroxypyridine	2.11
GMOLON4_3208	*acc*B	Biotin carboxyl carrier protein of acetyl-CoA carboxylase	ND
GMOLON4_3211	*uca*	Urea carboxylase	2.08
GMOLON4_3213	GMOLON4_3213	Hypothetical protein	ND
GMOLON4_3235	*nlp*D	Murein DD-endopeptidase MepM	2.20
GMOLON4_3237	*cas*9	CRISPR-associated endonuclease Cas9	3.52

Fold-change is calculated as the expression level of strain ON4^T cells growing in MMM over expression level of ON4^T cells growing in LB. Log2FC >2 was considered as different and represented up-regulated genes when ON4^T cells were growing in MMM;

ND, the normalized expression value in LB was 0 (not detected, i.e., reads mapping the particular gene were not detected), thus it was not possible to obtain the FC value;

*These genes were used in the validation of transcriptomic data

Table S7. Plasmid genes overexpressed in strain ON4^T growing in MMM over those growing in LB.

Locus Tag	Gene	Product	LOG2FC#
GMOLON4_3255	GMOLON4_3255	Permease	ND
GMOLON4_3256	GMOLON4_3256	hypothetical protein	ND
GMOLON4_3257	GMOLON4_3257	Prgl family	6.13
GMOLON4_3258	GMOLON4_3258	Transfer complex protein	8.51
GMOLON4_3259	*nlp*D	M23/M37 family peptidase	ND
GMOLON4_3260	GMOLON4_3260	Hypothetical protein	ND
GMOLON4_3261	GMOLON4_3261	ATPase;FHA family	ND
GMOLON4_3262	GMOLON4_3262	ATPase	5.32
GMOLON4_3263	GMOLON4_3263	hypothetical protein	ND
GMOLON4_3264	GMOLON4_3264	MaR family transcriptional regulator	ND
GMOLON4_3271	GMOLON4_3271		ND
GMOLON4_3273	GMOLON4_3273	hypothetical protein	ND
GMOLON4_3274	GMOLON4_3274	Nuclease (SNase domain-containing protein)	ND
GMOLON4_3275	GMOLON4_3275	Single-stranded DNA-binding protein	4.44
GMOLON4_3276	*par*A*	Plasmid partition protein homolog ParA	ND
GMOLON4_3278	GMOLON4_3278	hypothetical protein	ND
GMOLON4_3279	GMOLON4_3279	type II toxin-antitoxin interferase; sodium-indep. anion	ND
GMOLON4_0010	GMOLON4_0010	Transposase IS3/IS911 family protein	4.95
GMOLON4_0005	GMOLON4_0005	Transposase	ND
GMOLON4_3280	*mol*A*	Molinate hydrolase	15.21
GMOLON4_0020	GMOLON4_0020	Transposase IS3/IS911 family protein	7.61
GMOLON4_0015	GMOLON4_0015	Transposase	ND
GMOLON4_3281	*mol*A*	Molinate hydrolase	15.21
GMOLON4_0030	GMOLON4_0030	Transposase IS3/IS911 family protein	7.61
GMOLON4_0025	GMOLON4_0025	Transposase	ND
GMOLON4_3282	*mol*A*	Molinate hydrolase	15.21
GMOLON4_0040	GMOLON4_0040	Transposase IS3/IS911	7.60
GMOLON4_0035	GMOLON4_0035	Transposase	ND
GMOLON4_3283	GMOLON4_3283	Phospholipid carrier-dependent;glycosyltranferase	ND
GMOLON4_3284	GMOLON4_3284	alpha manosidase	ND
GMOLON4_3285	*fic*	Adenosine monophosphate-protein transferase VbhT	ND
GMOLON4_3286	GMOLON4_3286	Resolvase	ND
GMOLON4_3287	GMOLON4_3287	hypothetical protein	ND
GMOLON4_3288	GMOLON4_3288	hypothetical protein	ND
GMOLON4_3289	*mob*C	plasmid mobilization;relaxosome	ND
GMOLON4_3290	*rlx*	relaxase	ND
GMOLON4_3291	GMOLON4_3291	DUF3801;PcfB family	ND
GMOLON4_3292	*trs*K	TraG/TraD family	ND
GMOLON4_3293	GMOLON4_3293	hypothetical protein	ND

Fold-change is calculated as the expression level of strain ON4^T cells growing in MMM over expression level of ON4^T cells growing in LB. Log2FC >2 was considered as different and represented up-regulated genes when ON4^T cells were growing in MMM;

ND, the normalized expression value in LB was 0 (not detected, i.e., reads mapping the particular gene were not detected), thus it was not possible to obtain the FC value;

*These genes were used in the validation of transcriptomic data.

Table S8. Candidate genes involved in ACA mineralization and their respective closest neighbours for which experimental validation is described, based on BLASTp analysis (https://blast.ncbi.nlm.nih.gov/).

Step	Locus Tag	Gene	Product	LOG2FC#	Species	Query Cover (%)	Identity (%)	Accession	Substrate(s) degraded by the strain (experimental validation)	Reference
**I**	GMOLON4_435	*cin*	Nitric oxide synthase	5.85	*Leucobacter triazinivorans*	98	81.9	WP_130109160.1	prometryn (triazine)	[8]
					Rhodococcus jostii RHA1	100	45.2	NC_008268.1	mono/octa-chlorobiphenyls	[9]
	GMOLON4_436	*fpr*A	NADPH-ferredoxin reductase FprA	7.44	*Leucobacter triazinivorans*	100	71.6	WP_130109159.1	prometryn (triazine)	[8]
					Rhodococcus jostii RHA1	99	32.7	NC_008268.1	mono/octa-chlorobiphenyls	[9]
	GMOLON4_437	***bio*I**	Cytochrome P450	6.10	*M. smegmatis* mc^{2}155 (1999)	99	51.6	AAD28344.1	morpholine, piperidine, pyrrolidine	[10]
					Mycobacterium sp. RP1	97	51.0	CAC84231.1	morpholine	[11]
					Mycobacterium sp. HE5	97	51.0	AAV54064.1	pyridine, metyrapone, azole compounds	[12]
					Rhodococcus jostii RHA1	92	29.3	NC_008268.1	mono/octa-chlorobiphenyls	[9]
	GMOLON4_1038	***cyp*104**	Cytochrome P450-pinF2, plant-inducible	3.70	*Rhodococcus jostii* RHA1	96	32.7	NC_008268.1	mono/octa-chlorobiphenyls	[9]
					M. smegmatis mc^{2}155 (1999)	29	27.1	AAD28344.1	morpholine, piperidine, pyrrolidine	[10]
					Mycobacterium sp. HE5	29	26.5	AAV54064.1	pyridine, metyrapone, different azole compounds	[12]

Table S8. Continue

Step	Locus Tag	Gene	Product	LOG2FC #	Species	Query Cover (%)	Identity (%)	Accession	Substrate (experimental validation)	Reference
]I	GMOLON4_1039	GMOLON4 _1039	Hypothetical protein	1.19	*Mycobacterium* sp. RP1	20	27.3	CAC84233.1	morpholine	[11]
					Mycobacterium sp. HE5	20	27.3	AAV54066.1	pyridine, metyrapone, different azole compounds	[12]
	GMOLON4_3049	*cin*C	Cindoxin	1.79	*Rhodococcus jostii* RHA1	92	46.6	NC_008268.1	mono/octa-chlorobiphenyls	[9]
	GMOLON4_3050	*fpr*A	Oxidoreductase	1.86	*Rhodococcus jostii* RHA1	98	28.9	NC_008268.1	mono/octa-chlorobiphenyls	[9]
					Mycobacterium sp. RP1	36	23.8	CAC84233.1	morpholine	[11]
					Mycobacterium sp. HE5	36	23.8	AAV54066.1	pyridine, metyrapone, azole compounds	[12]
	GMOLON4_3051	***pip*A**	**Cytochrome P450**	1.72	*M. smegmatis* mc^2155 (1999)	99	53.0	AAD28344.1	morpholine, piperidine, pyrrolidine	[10]
					Mycobacterium sp. RP1	99	52.8	CAC84231.1	morpholine	[11]
					Mycobacterium sp. HE5	99	52.8	AAV54064.1	pyridine, metyrapone, different azole compounds	[12]
					Rhodococcus jostii RHA1	99	28.5	NC_008268.1	mono/octa-chlorobiphenyls	[9]
	GMOLON4_3097	***cpx*P**	**Cytochrome P450 monooxygenase**	3.10	*Rhodococcus jostii* RHA1	99	31.8	NC_008268.1	mono/octa-chlorobiphenyls	[9]
					M. smegmatis mc^2155 (1999)	69	29.9	AAD28344.1	morpholine, piperidine, pyrrolidine	[10]
					Mycobacterium sp. RP1	76	27.4	CAC84231.1	morpholine	[11]
					Mycobacterium sp. HE5	76	27.4	AAV54064.1	pyridine, metyrapone, different azole compounds	[12]

Table S8. Continue

Step	Locus Tag	Gene	Product	LOG2FC#	Species	Query Cover (%)	Identity (%)	Accession	Substrate(s) degraded by the strain (experimental validation)	Reference
]	GMOLON4_3098	GMOLON4_3098	Ferredoxin	ND	*Mycobacterium* sp. RP1	85	32.2	CAC84232.1	morpholine	[11]
					Mycobacterium sp. HE5	85	32.2	AAV54065.1	pyridine, metyrapone, different azole compounds	[12]
					M. smegmatis mc^{2}155 (1999)	85	36.1	Query_43195	morpholine, piperidine, pyrrolidine	[10]
	GMOLON4_3099	GMOLON4_3099	hypothetical protein	1.60	*Rhodococcus jostii* RHA1	96	29.0	NC_008268.1	mono/octa-chlorobiphenyls	[9]
					Mycobacterium sp. RP1	85	31.1	CAC84233.1	morpholine	[11]
					Mycobacterium sp. HE5	85	31.1	AAV54066.1	pyridine, metyrapone, different azole compounds	[12]
**II**	GMOLON4_2241	*cpn*A	Cyclopentanol dehydrogenase	2.44	*Comamonas* sp. NCIMB 9872	98	40.2	BAC22653.1	cyclopentanol	[17]
					Rhodococcus jostii RHA1	100	40.4	NC_008268.1	mono/octa-chlorobiphenyls	[9]
					Acinetobacter johnsonii	97	33.6	BAC80215.1	cyclopentanol	[18]
					Acinetobacter sp. strain NCIMB 9871	97	33.0	AB006902.2	cyclopentanol	[19]
					Rhodococcus sp. TK6	97	30.8	AAR27575.1	cyclohexanol, cyclohexanediols, hexanediols, cyclopentanol	[20]

Table S8. Continue

Step	Locus Tag	Gene	Product	LOG2FC#	Species	Query Cover (%)	Identity (%)	Accession	Substrate(s) degraded by the strain (experimental validation)	Reference
]IV	GMOLON4_1869	*gab*T	4-aminobutyrate aminotransferase	0.10	*Arthrobacter* sp. KI72	100	57.0	BDMH01000023.1	6-aminohexanoate	[21]
					Rhodococcus jostii RHA1	100	54.4	NC_008268.1	mono/octa-chlorobiphenyls	[9]
					Pseudomonas aeruginosa	94	43.2	WP_003106268.1	5-aminopentanoate	[22]
					Escherichia coli str. K-12 substr. MG1655	99	40.1	NC_000913.3	5-aminovaleriate	[13]
V	GMOLON4_913	*chn*E	6-oxohexanoate dehydrogenase	3.73	*Arthrobacter* sp. KI72	100	79.6	BDMH01000001.1	6-oxohexanoate	[21]
					Rhodococcus sp. Phi2	99	68.4	AAN37492.1	6-oxohexanoate	[24]
					Arthrobacter sp. BP2	95	65.7	Query_47643	6-oxohexanoate	[24]
					Escherichia coli str. K-12 substr. MG1655	99	40.4	NC_000913.3	glutamate semialdehyde	[13]
					Pseudomonas aeruginosa PAO1	99	33.1	NC_002516.2	5-oxopentanoic acid, succinate semialdehyde	[22]
					Acinetobacter sp. NCIMB 9871	91	31.1	Query_45701	6-oxohexanoate	[19]
					Pseudomonas aeruginosa PAO1	99	33.1	NC_002516.2	5-oxopentanoic acid, succinate semialdehyde	[22]
					Acinetobacter sp. NCIMB 9871	91	31.1	Query_45701	6-oxohexanoate	[19]
Fatty acid β-oxidation	GMOLON4_653	*fad*B	3-hydroxyacyl-CoA dehydrogenase	2.05	*Gulosibacter chungangensis*	99	86.4	WP_158051538.1		
					Gulosibacter faecalis	98	78.7	WP_019617459.1		
					Gulosibacter macacae	99	78.2	WP_124969129.1		
					Gulosibacter bifidus	99	69.2	WP_066055269.1		
					Pseudoclavibacter chungangensis	98	70.9	WP_158040990.1		

Table S8. Continue

Step	Locus Tag	*Gene*	Product	LOG2FC#	Species	Query Cover (%)	Identity (%)	Accession	Substrate(s) degraded by the strain (experimental validation)	Reference
Fatty acid β-oxidation	GMOLON4_885	*paa*J	3-oxoadipyl-CoA/3-oxo-5,6-dehydrosuberyl-CoA thiolase	4.18	*Leucobacter celer*	100	91.6	WP_053384615.1		
					Leucobacter weissii	100	86.5	WP_208095062.1		
					Leucobacter exalbidus	100	87.7	WP_209705628.1		
					Leucobacter tardus	100	87.5	WP_208235789.1		
					Microbacterium	99	85.9	WP_194398789.1		
	GMOLON4_886	*mmg*C	Acyl-CoA dehydrogenase MmgC	4.94	*Subtercola frigoramans*	97	76.7	WP_205106577.1		
					Subtercola frigoramans	97	75.6	WP_205112144.1		
					Microbacterium ginsengisoli	97	78.5	WP_045246911.1		
					Microbacterium	97	78.5	WP_005055001.1		
					Microbacterium	97	78.7	WP_135950157.1		
	GMOLON4_911	*mmg*B	Putative 3-hydroxybutyryl-CoA dehydrogenase	ND	*Arthrobacter citreus*	100	66.8	WP_152226206.1		
					Arthrobacter koreensis	100	65.3	WP_152273794.1		
					Arthrobacter mobilis	99	65.5	WP_168485169.1		
					Arthrobacter oryzae	100	64.9	WP_120951846.1		
					Arthrobacter pigmenti	99	65.5	WP_167995997.1		
	GMOLON4_912	*paa*A	Putative enoyl-CoA hydratase PaaA	3.59	*Microterricola pindariensis*	94	69.5	WP_104475409.1		
					Arthrobacter crystallopoietes	99	64.5	WP_074701139.1		
					Arthrobacter luteolus	98	66.8	WP_066296369.1		
					Arthrobacter citreus	98	66.0	WP_152226204.1		
					Rothia nasimurium	96	66.4	WP_185173893.1		
	GMOLON4_1457	*paa*H	Putative 3-hydroxybutyryl-CoA dehydrogenase PaaH	2.05	*Micrococcus terreus*	96	77.8	WP_091697831.1		
					Kocuria rhizophila	97	76.0	WP_135010697.1		
					Micrococcus terreus	95	77.9	WP_091699618.1		
					Micrococcus flavus	98	76.4	WP_135029386.1		
					Micrococcus lylae	95	77.7	WP_087133953.1		
	GMOLON4_1848	GMOLON4_1848	Short-chain dehydrogenase reductase 3a	2.14	*Streptomyces hirsutus*	100	80.1	WP_055593816.1		
					Microbacterium chengjingii	99	77.1	WP_166988594.1		
					Leucobacter japonicus	99	76.7	WP_053386641.1		
					Brachybacterium sacelli	100	75.8	WP_209905006.1		
					Herbiconiux flava	99	73.5	WP_179548088.1		

Table S8. Continue

Step	Locus Tag	*Gene*	Product	LOG2FC#	*Species*	Query Cover (%)	Identity (%)	Accession	Substrate(s) degraded by the strain (experimental validation)	Reference
**Fatty acid β-oxidation**	GMOLON4_2106	*acd*	Acyl-CoA dehydrogenase	3.49	*Paeniglutamicibacter terrestris*	97	69.7	WP_168150715.1		
					Gulosibacter molinativorax	99	68.2	WP_026935782.1		
					Microbacterium phyllosphaerae	100	67.5	WP_210098864.1		
					Subtercola frigoramans	97	65.6	WP_205106577.1		
					Subtercola frigoramans	97	66.5	WP_205112144.1		
	GMOLON4_2171	*fad*E	Putative acyl-CoA dehydrogenase	3.37	*Gulosibacter chungangensis*	99	82.7	WP_158052646.1		
					Gulosibacter macacae	97	74.3	WP_124971744.1		
					Gulosibacter bifidus	96	70.2	WP_066058106.1		
					Gulosibacter faecalis	98	71.3	WP_019617855.1		
					Gulosibacter sediminis	97	68.4	WP_193128009.1		
	GMOLON4_2554	*sfn*A	Acyl-CoA dehydrogenase/oxidase family protein	2.71	*Gulosibacter chungangensis*	93	83.4	WP_158051041.1		
					Subtercola frigoramans	92	65.5	WP_205107210.1		
					Acinetobacter	91	60.7	WP_004994108.1		
					Pseudomonas ovata	91	65.1	WP_109511643.1		
					Acinetobacter guillouiae	92	57.6	WP_096734473.1		
**N metabolism**	GMOLON4_438	glnA_2	Glutamine synthetase	6.29	*Leucobacter chironomi*	100	99.4	WP_024356860.1		
					Leucobacter muris	100	97.7	WP_128386488.1		
					Leucobacter viscericola	100	90.8	WP_166293206.1		
					Leucobacter celer	100	89.1	WP_083451410.1		
					Leucobacter triazinivorans	100	87.0	WP_130109157.1		
					Leucobacter luti	100	85.9	WP_130453621.1		
	GMOLON4_3052	glnA_2	Glutamine synthetase	2.19	*Gulosibacter chungangensis*	100	91.4	WP_158053315.1		
					Brevibacterium marinum	100	76.6	WP_167949181.1		
					Gulosibacter faecalis	98	70.7	WP_110463863.1		
					Gulosibacter macacae	98	70.4	WP_124970754.1		
					Gulosibacter bifidus	97	69.9	WP_083524475.1		

[#] Fold-change is calculated as the expression level of strain ON4^T cells growing in MMM over expression level of ON4^T cells growing in LB. Log2FC >2 was considered as different and represented up-regulated genes when ON4^T cells were growing in MMM

ND, the normalized expression value in LB was 0 (not detected, i.e., reads mapping the particular gene were not detected), thus it was not possible to obtain the FC value;

Table S9. Amino acid identity (%) of candidate genes involved on the degradation of heterocyclic- or aliphatic ring compounds with closest homologues harbored by *Gulosibacter* strains, based on Protein BLAST of the Basic Local Alignment Search Tool (https://blast.ncbi.nlm.nih.gov/).

Locus Tag	Gene	Product	Query Cover (%)	Identity (%)	Accession	Description	Strain
GMOLON4_3280 /3281/3282	*mol*A	Molinate hydrolase	86	21.8	WP_066059557.1	imidazolonepropionase	*G. bifidus* NBRC 103089^{T}
			19	31.1	WP_158051577.1	dihydroorotase	*G. chungangensis* KCTC 13959^{T}
			21	28.4	WP_019617502.1	dihydroorotase	*G. faecalis* ATCC 13722^{T}
			14	34.8	WP_201517484.1	D-aminoacylase	*G. hominis* 401352-2018^{T}
			21	32.1	WP_201520781.1	dihydroorotase	*G. hominis* 401468-2018
			21	32.0	WP_201613561.1	dihydroorotase	*G. hominis* 404866-2018
			20	32.6	WP_124971201.1	dihydroorotase	*G. macacae* YIM 102482-1^{T}
			29	25.9	WP_125106610.1	amidohydrolase	"*G.massiliensis*" Marseille-P7157^{T}
			19	28.9	WP_193127795.1	dihydroorotase	*G. sediminis* YIM M12148^{T}
			19	30.0	WP_249385770.1	dihydroorotase	*G. sediminis* ACHW.36C
			27	34.9	WP_176386767.1	amidohydrolase family protein	*Gulosibacter* sp. strain 10
GMOLON4_437	*bio*I	Cytochrome P450	97	56.1	WP_066056981.1	cytochrome P450	*G. bifidus* NBRC 103089^{T}
			100	45.7	WP_158053314.1	cytochrome P450	*G. chungangensis* KCTC 13959^{T}
			100	71.6	WP_019619516.1	cytochrome P450	*G. faecalis* ATCC 13722^{T}
			NSS	NSS	NSS	NSS	*G. hominis* 401352-2018^{T}
			99	46.2	WP_201522186.1	FAD-dependent oxidoreductase	*G. hominis* 401468-2018
			NSS	NSS	NSS	NSS	*G. hominis* 404866-2018
			100	69.2	WP_124970757.1	cytochrome P450	*G. macacae* YIM 102482-1^{T}
			98	45.9	WP_164509754.1	FAD-dependent oxidoreductase	"*G.massiliensis*" Marseille-P7157^{T}
			100	70.9	WP_193128114.1	cytochrome P450	*G. sediminis* YIM M12148^{T}
			100	70.9	WP_249386280.1	cytochrome P450	*G. sediminis* ACHW.36C
			80	28.0	WP_087004166.1	cytochrome P450	*Gulosibacter* sp. strain 10

Table S9. Continue

Locus Tag	Gene	Product	Query Cover (%)	Identity (%)	Accession	Description	Strain
GMOLON4_3051	*pip*A	Cytochrome P450	97	58.2	WP_066056981.1	cytochrome P450	*G. bifidus* NBRC 103089^{T}
			100	90.8	WP_158053314.1	cytochrome P450	*G. chungangensis* KCTC 13959^{T}
			98	82.2	WP_019619516.1	cytochrome P450	*G. faecalis* ATCC 13722^{T}
			NSS	NSS	NSS	NSS	*G. hominis* 401352-2018^{T}
			98	78.9	WP_201522187.1	cytochrome P450	*G. hominis* 401468-2018
			97	28.0	WP_201518270.1	FAD-dependent oxidoreductase	*G. hominis* 404866-2018
			99	80.1	WP_124970757.1	cytochrome P450	*G. macacae* YIM 102482-1^{T}
			98	81.0	WP_125107001.1	cytochrome P450	"*G.massiliensis*" Marseille-P7157^{T}
			98	80.7	WP_193128114.1	cytochrome P450	*G. sediminis* YIM M12148^{T}
			98	80.2	WP_249386280.1	cytochrome P450	*G. sediminis* ACHW.36C
			73	26.9	WP_087004166.1	cytochrome P450	*Gulosibacter* sp. strain 10
GMOLON4_2241	*cpn*A	Cyclopentanol dehydrogenase	41	50.1	WP_066056978.1	FAD-dependent oxidoreductase	*G. bifidus* NBRC 103089^{T}
			77	39.7	WP_158053376.1	SDR family oxidoreductase	*G. chungangensis* KCTC 13959^{T}
			77	37.1	WP_019618924.1	glucose 1-dehydrogenase	*G. faecalis* ATCC 13722^{T}
			78	33.0	WP_201517822.1	glucose 1-dehydrogenase	*G. hominis* 401352-2018^{T}
			78	33.5	WP_201520994.1	glucose 1-dehydrogenase	*G. hominis* 401468-2018
			78	33.3	WP_201613262.1	glucose 1-dehydrogenase	*G. hominis* 404866-2018
			77	43.5	WP_124968772.1	glucose 1-dehydrogenase	*G. macacae* YIM 102482-1^{T}
			77	35.3	WP_125107678.1	SDR family oxidoreductase	"*G.massiliensis*" Marseille-P7157^{T}
			77	35.3	WP_193127701.1	SDR family oxidoreductase	*G. sediminis* YIM M12148^{T}
			77	35.6	WP_249387962.1	SDR family oxidoreductase	*G. sediminis* ACHW.36C
			77	50.4	WP_087009159.1	glucose 1-dehydrogenase	*Gulosibacter* sp. strain 10

Table S9. Continue

Locus Tag	Gene	Product	Query Cover (%)	Identity (%)	Accession	Description	Strain
GMOLON4_1492	*chn*B	Cyclohexanone monooxigenase	77	30.8	WP_066058705.1	NAD(P)/FAD-dependent oxidoreductase	*G. bifidus* NBRC 103089^{T}
			98	39.0	WP_158051123.1	NAD(P)/FAD-dependent oxidoreductase	*G. chungangensis* KCTC 13959^{T}
			34	24.0	WP_019619687.1	NAD(P)/FAD-dependent oxidoreductase	*G. faecalis* ATCC 13722^{T}
			91	29.1	WP_230972893.1	NAD(P)/FAD-dependent oxidoreductase	*G. hominis* 401352-2018^{T}
			91	29.1	WP_230972694.1	NAD(P)/FAD-dependent oxidoreductase	*G. hominis* 401468-2018
			91	29.1	WP_230972694.1	NAD(P)/FAD-dependent oxidoreductase	*G. hominis* 404866-2018
			87	27.4	WP_124969253.1	NAD(P)/FAD-dependent oxidoreductase	*G. macacae* YIM 102482-1^{T}
			38	22.8	WP_206427704.1	NAD(P)/FAD-dependent oxidoreductase	"*G.massiliensis*" Marseille-P7157^{T}
			14	29.9	WP_235988324.1	NAD(P)-binding domain-containing protein	*G. sediminis* YIM M12148^{T}
			14	32.5	WP_249386932.1	NAD(P)-binding domain-containing protein	*G. sediminis* ACHW.36C
			96	25.6	WP_087005455.1	NAD(P)/FAD-dependent oxidoreductase	*Gulosibacter* sp. strain 10
GMOLON4_1493	*chn*C	Caprolactone hydrolase	37	37.5	WP_066058708.1	alpha/beta hydrolase	*G. bifidus* NBRC 103089^{T}
			87	32.6	WP_158053219.1	alpha/beta hydrolase fold domain-containing protein	*G. chungangensis* KCTC 13959^{T}
			69	29.4	WP_019617618.1	alpha/beta hydrolase	*G. faecalis* ATCC 13722^{T}
			36	38.1	WP_201518604.1	alpha/beta hydrolase	*G. hominis* 401352-2018^{T}
			36	37.3	WP_201521913.1	alpha/beta hydrolase	*G. hominis* 401468-2018

Table S9. Continue

Locus Tag	Gene	Product	Query Cover (%)	Identity (%)	Accession	Description	Strain
GMOLON4_1493	*chn*C	Caprolactone hydrolase	36	38.1	WP_201614442.1	alpha/beta hydrolase	*G. hominis* 404866-2018
			51	32.9	WP_124969251.1	alpha/beta hydrolase	*G. macacae* YIM 102482-1^T
			25	33.7	WP_164509703.1	MULTISPECIES: carboxylesterase/lipase family protein	"*G.massiliensis*" Marseille-P7157^T
			25	33.7	WP_164509703.1	MULTISPECIES: carboxylesterase/lipase family protein	*G. sediminis* YIM M12148^T
			18	40.0	WP_249387065.1	aldo/keto reductase	*G. sediminis* ACHW.36C
			69	29.9	WP_087007226.1	alpha/beta hydrolase	*Gulosibacter* sp. strain 10
GMOLON4_1869	*gab*T	4-aminobutyrate aminotransferase	100	74.9	WP_066055799.1	4-aminobutyrate--2-oxoglutarate transaminase	*G. bifidus* NBRC 103089^T
			100	90.3	WP_158053479.1	4-aminobutyrate--2-oxoglutarate transaminase	*G. chungangensis* KCTC 13959^T
			100	79.4	WP_019619747.1	4-aminobutyrate--2-oxoglutarate transaminase	*G. faecalis* ATCC 13722^T
			100	80.3	WP_201521238.1	4-aminobutyrate--2-oxoglutarate transaminase	*G. hominis* 401352-2018^T
			100	80.1	WP_201517373.1	4-aminobutyrate--2-oxoglutarate transaminase	*G. hominis* 401468-2018
			100	80.1	WP_201613963.1	4-aminobutyrate--2-oxoglutarate transaminase	*G. hominis* 404866-2018
			99	76.8	WP_124969570.1	4-aminobutyrate--2-oxoglutarate transaminase	*G. macacae* YIM 102482-1^T
			100	76.8	WP_125107799.1	4-aminobutyrate--2-oxoglutarate transaminase	"*G.massiliensis*" Marseille-P7157^T
			89	31.0	WP_125106693.1	MULTISPECIES: ornithine--oxo-acid transaminase	*G. sediminis* YIM M12148^T

Table S9. Continue

Locus Tag	Gene	Product	Query Cover (%)	Identity (%)	Accession	Description	Strain
GMOLON4_1869	*gab*T	4-aminobutyrate aminotransferase	100	76.8	WP_249387210.1	4-aminobutyrate--2-oxoglutarate transaminase	*G. sediminis* ACHW.36C
			100	82.8	WP_087007173.1	4-aminobutyrate--2-oxoglutarate transaminase	*Gulosibacter* sp. strain 10
GMOLON4_913	*chn*E	6-oxohexanoate dehydrogenase	98	49.6	WP_066055442.1	NAD-dependent succinate-semialdehyde dehydrogenase	*G. bifidus* NBRC 103089^{T}
			98	55.2	WP_158053317.1	NAD-dependent succinate-semialdehyde dehydrogenase	*G. chungangensis* KCTC 13959^{T}
			98	48.3	WP_019617979.1	NAD-dependent succinate-semialdehyde dehydrogenase	*G. faecalis* ATCC 13722^{T}
			98	49.7	WP_201518840.1	NAD-dependent succinate-semialdehyde dehydrogenase	*G. hominis* 401352-2018^{T}
			98	49.7	WP_201521132.1	NAD-dependent succinate-semialdehyde dehydrogenase	*G. hominis* 401468-2018
			98	49.7	WP_201521132.1	NAD-dependent succinate-semialdehyde dehydrogenase	*G. hominis* 404866-2018
			98	56.8	WP_124970736.1	NAD-dependent succinate-semialdehyde dehydrogenase	*G. macacae* YIM 102482-1^{T}
			98	46.8	WP_125106382.1	MULTISPECIES: aldehyde dehydrogenase family protein	"*G.massiliensis*" Marseille-P7157^{T}
			98	46.8	WP_125106382.1	MULTISPECIES: aldehyde dehydrogenase	*G. sediminis* YIM M12148^{T}
			98	47.4	WP_249386986.1	aldehyde dehydrogenase family protein	*G. sediminis* ACHW.36C
			98	56.1	WP_087007928.1	NAD-dependent succinate-semialdehyde dehydrogenase	*Gulosibacter* sp. strain 10

NSS, No significant similarity found.

Table S10. Characteristics of primers targeting the genes used for the validation of the transcriptomic data, plasmid copy number and the 16S rRNA and *mol*A genes copy number.

Predicted gene product	Symbol	Locus Tag	Location	Primer name	Primer sequence (5'-3')	Position	Primer name	Primer sequence (5'-3')	Position
**Reference genes**									
DNA gyrase subunit A	*gyr*A	2427	contigA	gyrA_qF	ATGTGGTCCCCATCCTTGA	2601894	gyrA_F	AACCGACTGGTCAGACTCAA	2601368
				gyrA_qR	TCTCGCTCTACGACACGAAC	2601996	gyrA_R	CCCGTTACACCGAGACAAAG	2603526
DNA gyrase subunit B	*gyr*B	2428	contigA	gyrB_qF	ACAGTACCCAGCGAGAGA	2605874	gyrB_F	GTTCATCGTCGTGTCCCAGA	2604128
				gyrB_qR	GACTAGAAGCGGTTCGTAAGC	2605995	gyrB_R	ACAAGGTGGAAAACCAGTATGG	2606043
Protein RecA	*rec*A	327	contigA	recA_qF	CTCAACACCAGCAAGACAACC	315358	recA_F	CGGAATCCTCAGGTAAGACGA	315014
				recA_qR	CGCACCGAAGCATAGAACTTG	315479	recA_R	GCGGAGCCATCTTGTTCTTC	315570
16S rRNA [#]	16S	373; 1890; 2008	contigA	16S_qF	TTGGTTCTGGATCGGCTCAC	368593; 1988126; 2132910	27F	GAGTTTGATCCTGGCTCAG	368385; 1988334; 2133118
				16S_qR	GCAATATTCCCCACTGCTGC	368741; 1987978; 2132761	1492R	TACCTTGTTACGACTT	369866; 1986854; 2131637
**Target genes**									
Molinate hydrolase	*mol*A	3280; 3281; 3282	contigB	F10*	ACGATCGCGATTGTCGGCGG	19754; 22786; 25799	F10*	ACGATCGCGATTGTCGGCGG	19754; 22786; 25799
				R310	GGCTTCCTCGATGACCTTG	20038; 23051; 26064	R1122*	ATCCACACGAAGTGGTCCTC	20865; 23878; 26891
5-methyltetrahydropteroyltriglutamate-homocysteine methyltransferase	*met*E	1539	contigA	Msynt_qF	AGGAGCGGGAACGATTCT	1604541	Msynt_F	GTTACCGTGACCAGTTCCTC	1604235
				Msynt_qR	TGTTACGGCTACGGGATTAAG	1604629	Msynt_R	GAACATGAACACGCTTTTGCCG	1605269

Table S10. Continue

Predicted gene product	Symbol	Locus Tag	Location	Primer name	Primer sequence (5'-3')	Position	Primer name	Primer sequence (5'-3')	Position
**Target genes**									
Caprolactone hydrolase	*chn*C	1493	contigA	Estlip_qF	CGGCGAGAAGACGATTACCT	1555633	Estlip_F	GTTGTTCGTCTTGAGGGTCT	1555588
				Estlip_qR	CCCGAACAACATCACGACCAT	1555750	Estlip_R	CTATCAAGTCGGACGCACTA	1556156
Nitric oxide synthase	*cin*C	435	contigA	Flav_qF	CCTGGCTGTACGTCTCTTCA	435452	Flav_F	GCCCATGTCCCATGAAGAGG	435360
				Flav_qR	GAGATCTACCCAACACGGCG	435577	Flav_R	AGTTCACGGTTCTCTACGGC	435754
Alcohol dehydrogenase	*adh*P	1496	contigA	Zn_qF	TGCGTGCTGTTGACGAAATC	1559922	Zn_F	CCATCTTCCATTGCGGTTAGT	1559681
				Zn_qR	GGTGATGAGACCAACGGTT	1560004	Zn_R	GGGTTCCTGTCGTTGCTT	1560526
NADPH-ferredoxin reductase FprA	*fpr*A	436	contigA	NADPH_qF	TCGTGTTGAAAGAGCCTGTCG	436888	NADPH_F	GAAGCCGAGTGTAGACGG	435978
				NADPH_qR	GCTGCGAACTCACCATCTTC	437011	NADPH_R	CTGCTATCTCGCTCAGTCG	437049
Hydantoin utilization protein A	*hyu*A	3230	contigA	Nhyd_qF	CGCTGATTCGGATGGGATT	3407874	Nhyd_F	ACAAATGCGGGTAACTAAAGTG	3406377
				Nhyd_qR	GCTCATACTGCTCGTGGAA	3408010	Nhyd_R	GGATGCGGATATTAAGCCAT	3408322
Plasmid partition protein homolog ParA	*par*A	3276	contigB	parA_qF	AGCTCCCGATTCTTACCCCA	17043	parA_F	ACATCACCAGCGTTCTCACC	16536
				parA_qR	AGACATGAGCAGTTCCCTTC	17201	parA_R	GGTTACTCCCCTACTCCATC	17604

* Primer from Duarte et al. [25]

# This set of primers was used only for determining the number of copies of the plasmid and not as a reference gene for transcriptome validation.

Table S11. qPCR characteristics for the analysed genes.

Symbol	Predicted gene product	Expected product size (bp)	Primer concentration (nM)	DMSO (1 µL/reaction)	Slope	Efficiency (%)	Amplification
**Reference genes**							
*gyr*A	DNA gyrase subunit A	103	200	Y	-3.449	94.96	1.950
*gyr*B	DNA gyrase subunit B	122	200	Y	-3.777	83.98	1.840
*rec*A	Protein RecA	122	200	N	-3.387	97.35	1.974
16S	16S rRNA	153	200	Y	-3.559	90.98	1.910
**Target genes**							
*mol*A	Molinate hydrolase	285	200	N	-3.573	90.49	1.905
*met*E	5-methyltetrahydropteroyltriglutamate-homocysteine methyltransferase	89	200	N	-3.505	92.89	1.929
*chn*C	Caprolactone hydrolase	118	300	N	-3.704	86.20	1.865
*cin*C	Nitric oxide synthase	126	400	Y	-3.611	89.21	1.892
*adh*P	Alcohol dehydrogenase	83	300	Y	-3.77	84.17	1.842
*fpr*A	NADPH-ferredoxin reductase FprA	124	200	Y	-3.722	85.64	1.856
*hyu*A	Hydantoin utilization protein A	137	200	Y	-3.689	86.67	1.867
*par*A	Plasmid partition protein homolog ParA	159	200	N	-3.667	87.37	1.874

Y, yes; N, no.

Table S12. Bacterial strains and plasmids used in the present study for the heterologous expression assays.

Strain or Plasmid	Description	Source or Reference
**Strains**		
E. coli JM109	*recA1 endA1 gyr96A thi hsdR17 supE44 relA1* lambda$^-$ Delta*(lac-proAB) (F' traD36 proAB laqI^{q}ZDeltaM15)*	DSM 3423
E. coli BL21 (DE3) pLysS	F-*omp*T *hsd*SB (rB$^-$, mB$^-$) *galdcmrne*131 (DE3) pLysS (Km^R)	[26,27]
**Plasmids**		
pTZ57R/T	Linear vector, ddT tailed, cloning vector, Amp^r	Thermo Fisher Scientific
pTZ57R/T-*chn*C	pTZ57R/T with *chnC* gene	This study
pTZ57R/T-*hyu*A/B	pTZ57R/T with *hyuA* and *hyuB* genes	This study
pET-30b(+)	His•Tag, S•Tag, expression vector, Km^r	Novagen
pET-*chn*C	pET-30b(+) (*EcoRI*/*NdeI*) with *chnC* gene	This study
pET-*hyu*A/B	pET-30b(+) (*NdeI*/*SalI*) with *hyuA* and *hyuB* genes	This study

Amp^r: ampicillin resistance; Km^r: kanamycin resistance; *chnC*: Caprolactone hydrolase; *hyuA* and *hyuB*: Hydantoin utilization protein A and Hydantoinase B.

Table S13. Description of the primers used to amplify the complete sequence of *chnC* and *hyuA/hyuB* genes.

Predicted gene product	Symbol	Restriction Enzymes	Primer name	Primer sequence (5'-3')	Product length (bp)	Reference
Caprolactone hydrolase	*chnC*	*Eco*RI	chnC-EcoRI Fw	TAACCGAATTCGTTACTATCGTGCCGAGCCGTACCA	933	This study
		*Nde*I	chnC- NdeI Rv	TGGCATATGACCAGCAAC ATTGCTATCAAG		This study
Hydantoin utilization protein A and Hydantoinase B	*hyuA/hyuB*	*Nde*I	hyuA-NdeI Fw	TGGCATATGAGCAACATT CGTGTTGCAGT	4046	This study
		*Sal*I	hyuB SalI Rv	TATGTCGACATTCTACGA CTTCCAGTGGCCAG		This study

Table S14. Description of the primers used to screen clones.

Primer name	Primer sequence (5'-3')	Plasmid	Annealing Temperature	Reference
M13F-pUC	GTTTTCCCAGTCACGAC	pTZ57R/T	56	Promega
M13R-pUC	CAGGAAACAGCTATGAC			
T7 Promoter	TAATACGACTCACTATAGGG	pET-30b(+)		Novagen
T7 Terminator	GCTAGTTATTGCTCAGCGG			

Table S15. Description of the primers used to amplify the 16S rRNA and 23S rRNA genes from strain $ON4^T$.

Primer	Sequence 5´- 3'	Target gene	Reference
27F	GAGTTTGATCCTGGCTCAG	16S rRNA	[28]
1525HS	GCCAGTGAATTGTAATACGACTCACTATAGGGAAGGAGGTGATCCAGCC		[29,30]
256F	AGTAGTGGCGAGCGAA	23S rRNA	[30]*
2490HS	GCCAGTGAATTGTAATACGACTCACTATAGGGCGACATCGAGGTGCCAAA		[29,30]

*the degenerated primer described by Hunt et al. [30] was modified in order to be specific for strain $ON4^T$;

HS (reverse primers were modified to contain the T7 RNA polymerase promoter sequence [29,30]

Query	Subject	Identity	length	mismatches	indels	Query start	Query end	Subject start	Subject end	E value	Bitscore
contigA	contig00013__length_84892	100	63611	0	0	3402048	3465658	84892	21282	0	1.15E+05
contigA	contig00013__length_84892	100	21281	0	0	1	21281	21281	1	0	38378

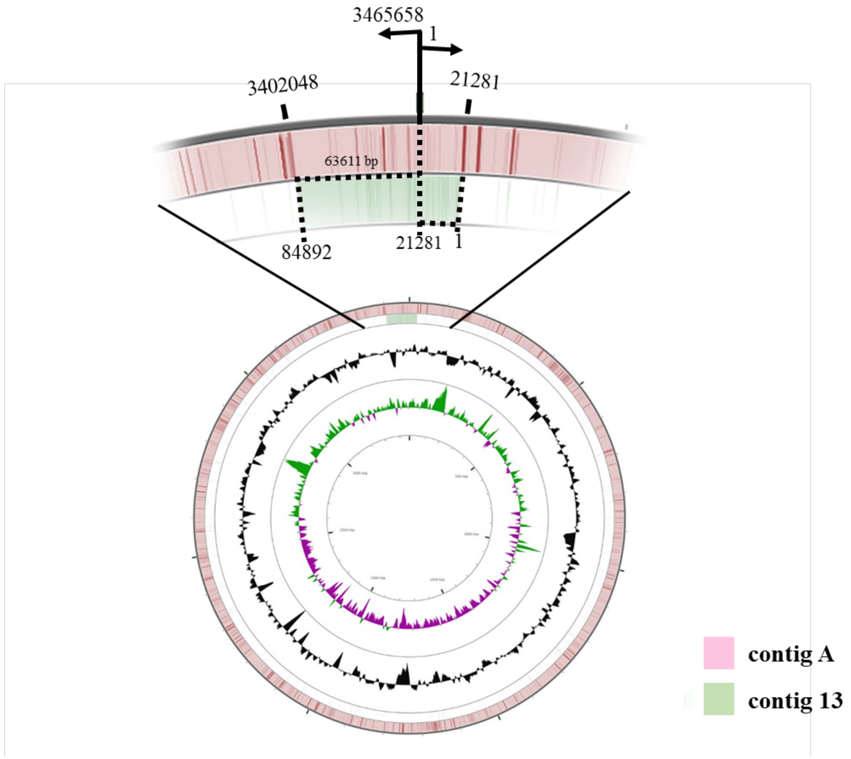

Figure S1. BLASTn of contig A (ON4$^{T}_{PacBio}$ CP028426) with contig 13 (ON4$^{T}_{454}$ PXVE00000000) genome using Contiguity (http://mjsull.github.io/Contiguity/) and the genome visualization software (http://stothard.afns.ualberta.ca/cgview_server/).

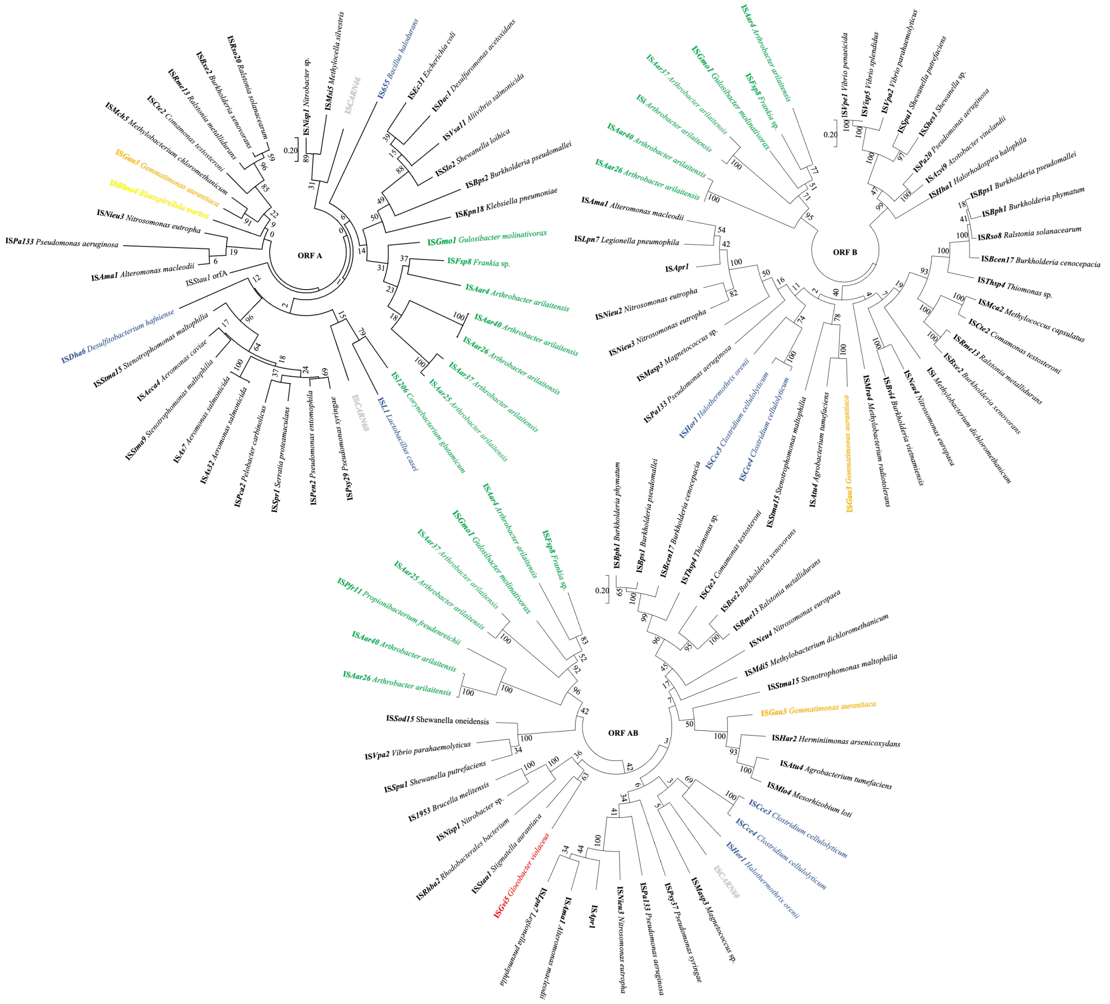

Figure S2. Dendrogram based on the alignment of the amino acid sequences of ORFA, ORFB and the predicted ORFAB of the DDE transposase of ISGmo1 and their forty closest sequences available at the ISfinder database. The sequences belonging to microorganisms affiliated to phylum *Actinobacteria* are in green, to *Firmicutes* in blue, to *Proteobacteria* in black, to *Gemmatimonadetes* in orange, to *Planctomycetes* in yellow, to *Cyanobacteria* in red, and those retrieved from metagenomic data are in grey.

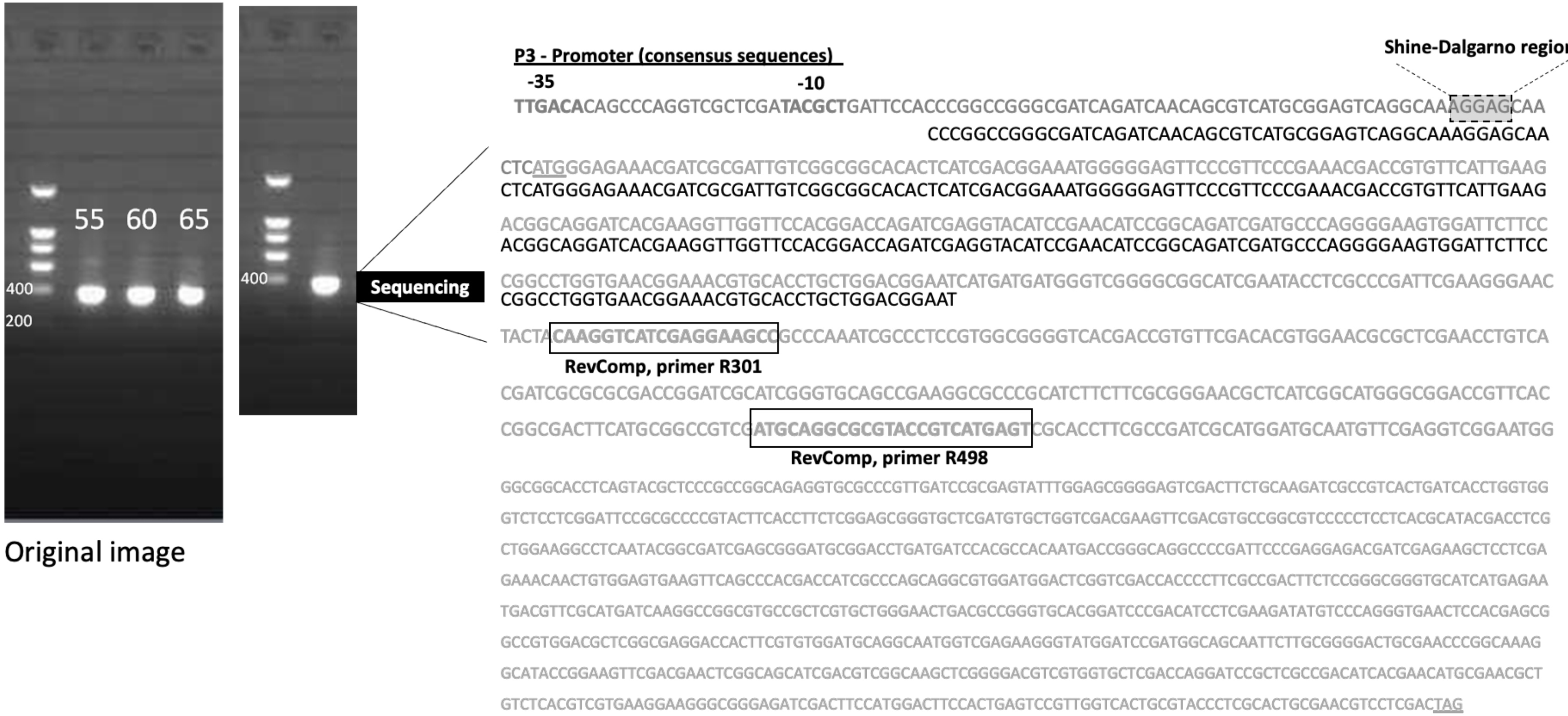

Figure S3. Characterization of the *mol*A regulatory region based on 5’RACE and ON4^T genome sequencing (ON4$^T_{PacBio}$ CP028427).

Bold grey letters, -35 and -10 consensus sequences of the promoter (corresponds to P3, Figure 2); Dashed line box, Shine - Dalgarno sequence; Black lined boxes, Reverse primers of the 1st and 2nd PCR of the 5´RACE. Underlined ATG, TAG, *mol*A gene start and stop codon, respectively; Black letters, sequence of the 5´RACE amplicon.

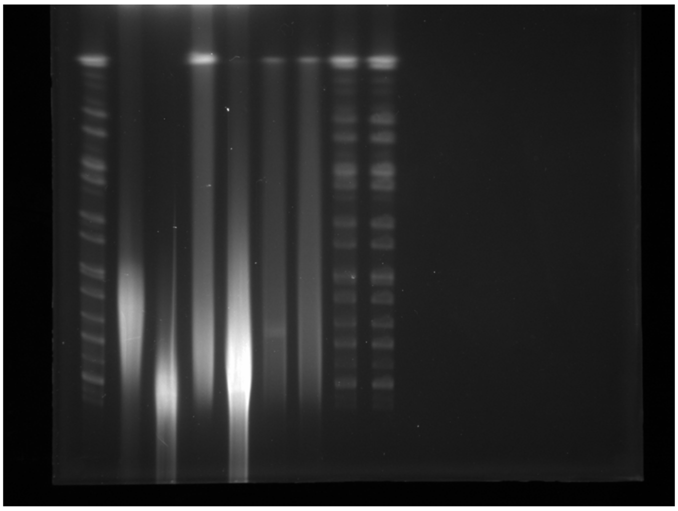

Original image

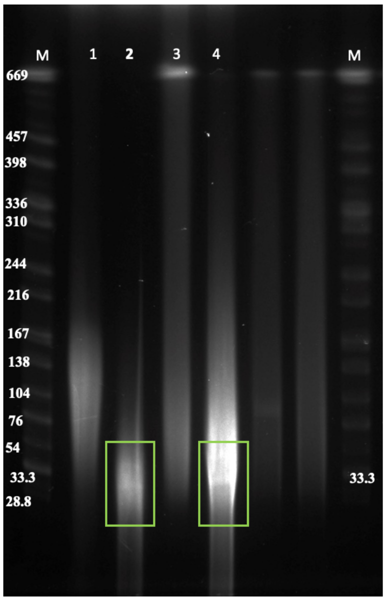

Figure S4. Pulsed-Field Gel Electrophoresis (PFGE) fingerprinting patterns of *G. molinativorax* ON4^T DNA non-digested (lane 1 and 3) and digested with S1 nuclease (lane 2 and 4). PFGE marker (M) (*Salmonella* serovar Braenderup H9812) digested with the *Xba*I enzyme [31].

Growth in MMM

Growth in LBM

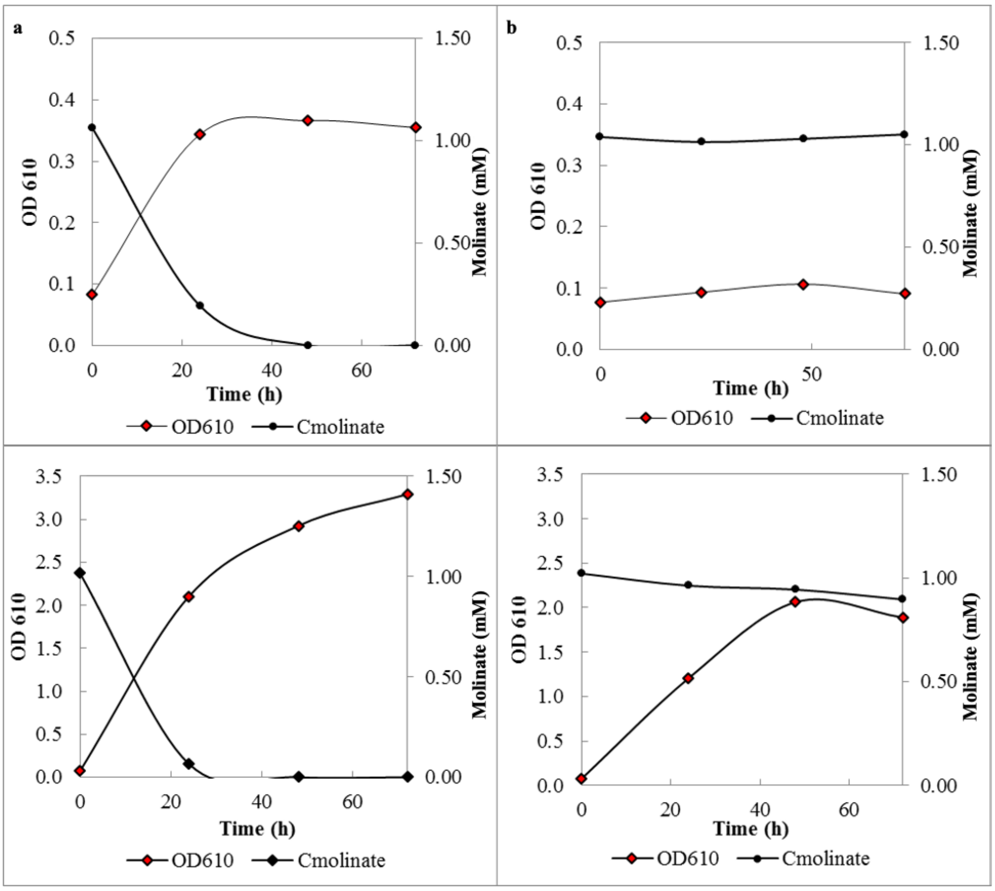

Figure S5. Molinate degradation by *G. molinativorax* strains ($ON4^T$ and $ON4^{(-)}$) over a 72 h incubation period in mineral medium B [1] supplemented with 1 mM molinate and 0.2 g/L yeast extract (MMM) and in Luria-Bertani Broth supplemented with 1 mM molinate (LBM). **a.** *G. molinativorax* strain $ON4^T$ **b.** *G. molinativorax* strain $ON4^{(-)}$.

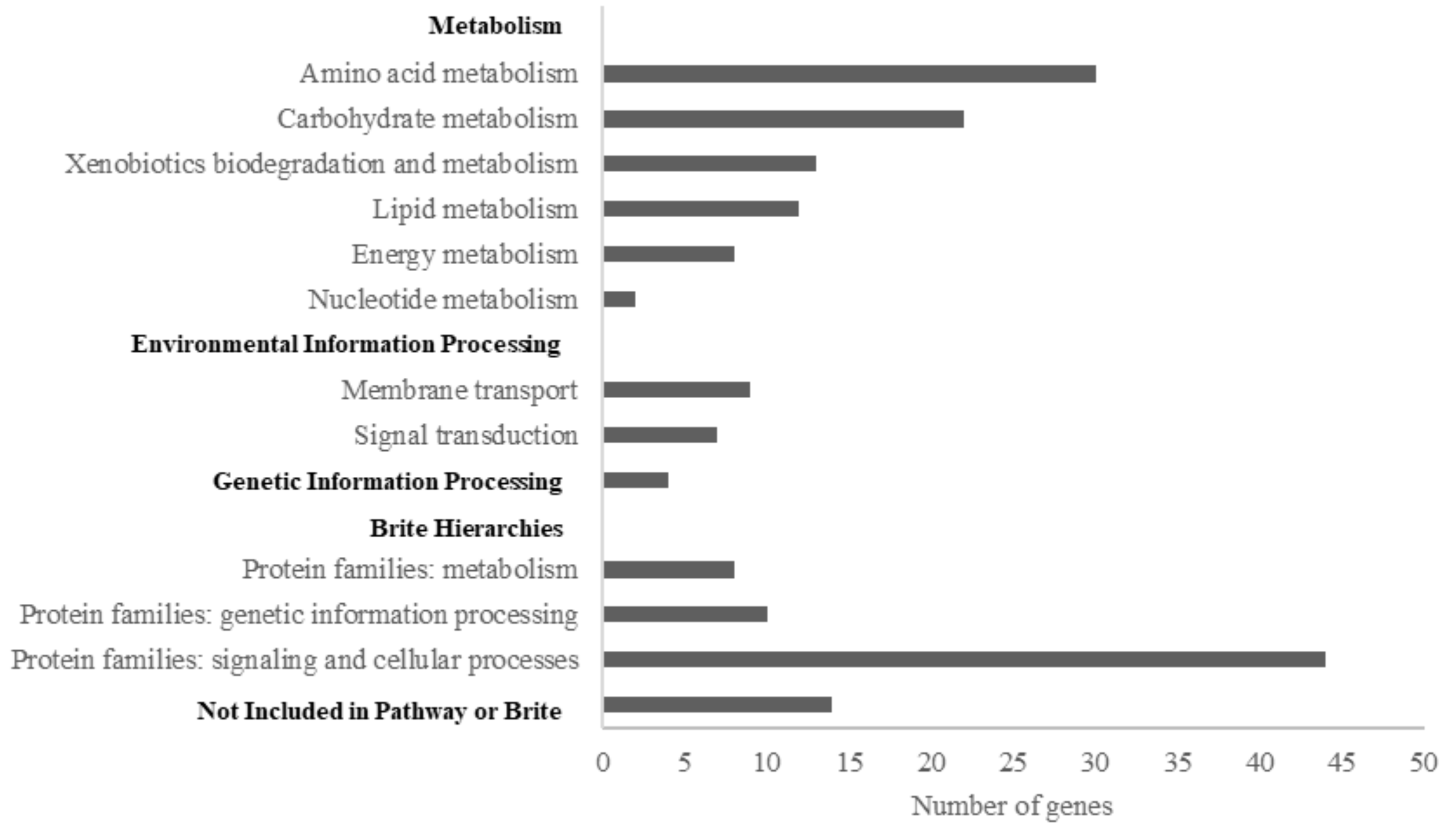

Figure S6. Functional categorization of the genes overexpressed in strain $ON4^T_{MMM}$ according to KEGG Orthology (KO) categories.

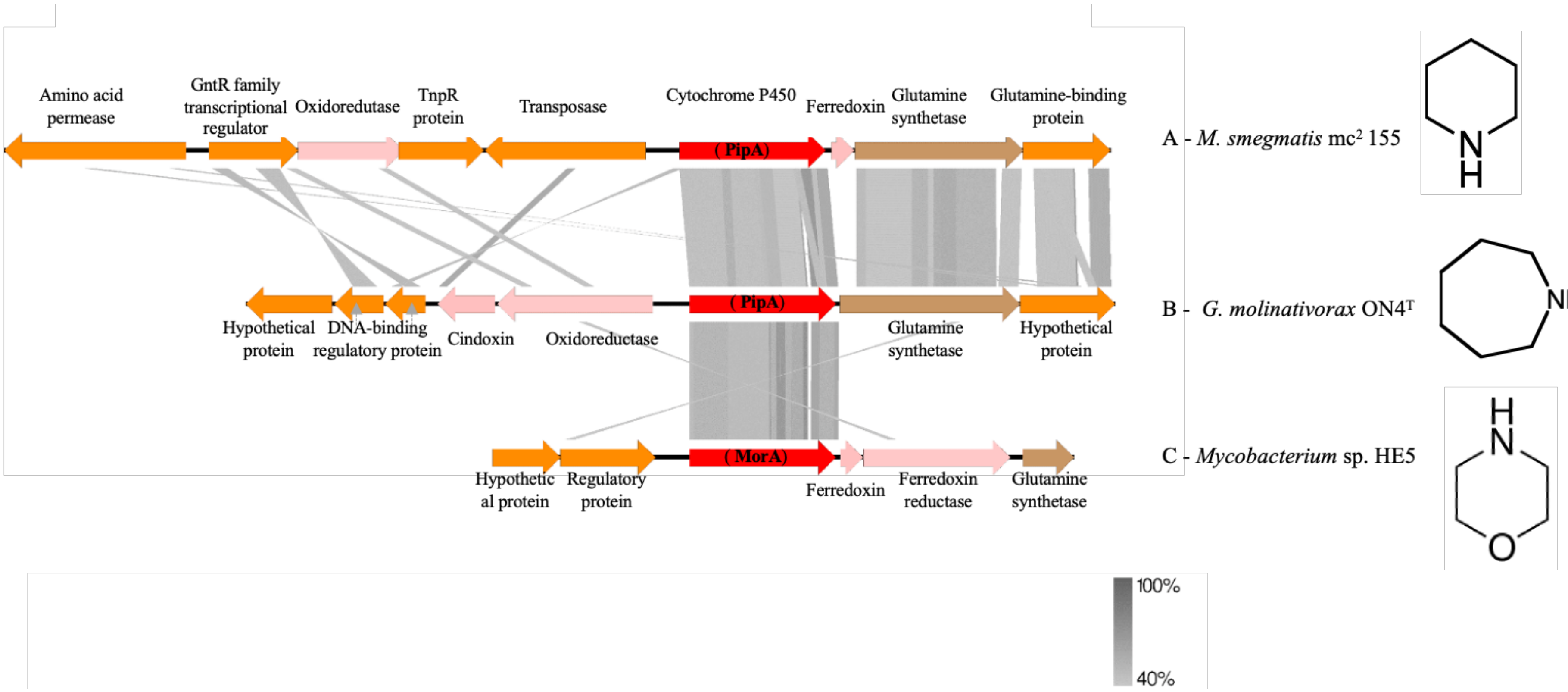

Figure S7. BLASTx between genes in the vicinity of cytochrome P450 (*pip*A) gene from strain ON4^T and those in the vicinity of cytochrome P450 gene involved in piperidine degradation by *Mycobacterium smegmatis* mc^{2}155 [10] (accession number CP009494) and involved in the conversion of morpholine by *Mycobacterium* sp. HE5 [12] (accession number AY816211), using Easyfig software [31].

References

1. Barreiros, L. *et al.* A novel pathway for mineralization of the thiocarbamate herbicide molinate by a defined bacterial mixed culture. *Environ. Microbiol.* **5**, 944–953 (2003).
2. Kumar, S., Stecher, G. & Tamura, K. MEGA7: Molecular evolutionary genetics analysis version 7.0 for bigger datasets. *Mol. Biol. Evol.* **33**, 1870–1874 (2016).
3. Larkin, M. A. *et al.* Clustal W and Clustal X version 2.0. *Bioinformatics* **23**, 2947–2948 (2007).
4. Stewart, F. J., Ottesen, E. A. & Delong, E. F. Development and quantitative analyses of a universal rRNA-subtraction protocol for microbial metatranscriptomics. *ISME J.* **4**, 896–907 (2010).
5. Yoon, S. H., Ha, S. min, Lim, J., Kwon, S. & Chun, J. A large-scale evaluation of algorithms to calculate average nucleotide identity. *Antonie van Leeuwenhoek, Int. J. Gen. Mol. Microbiol.* **110**, 1281–1286 (2017).
6. Abby, S. S. *et al.* Identification of protein secretion systems in bacterial genomes. *Sci. Rep.* **6**, 1–14 (2016).
7. Li, X. *et al.* oriTfinder: a web-based tool for the identification of origin of transfers in DNA sequences of bacterial mobile genetic elements. *Nucleic Acids Res.* **46**, W229–W234 (2018).
8. Sun, L.-N. *et al.* *Leucobacter triazinivorans* sp. nov., a *s*-triazine herbicide prometryn-degrading bacterium isolated from sludge. doi:10.1099/ijsem.0.002483.
9. Takeda, H. *et al.* Dual two-component regulatory systems are involved in aromatic compound degradation in a polychlorinated-biphenyl degrader, *Rhodococcus jostii* RHA1. *J. Bacteriol.* **192**, 4741–4751 (2010).
10. Poupin, P., Ducrocq, V., Hallier-Soulier, S. & Truffaut, N. Cloning and characterization of the genes encoding a cytochrome P450 (PipA) involved in piperidine and pyrrolidine utilization and its regulatory protein (PipR) in *Mycobacterium smegmatis* mc^{2}155. *J. Bacteriol.* **181**, 3419–3426 (1999).
11. Besse, P. *et al.* Degradation of morpholine and thiomorpholine by an environmental *Mycobacterium* involves a cytochrome P450. Direct evidence of intermediates by *in situ* ^{1}H NMR. *J. Mol. Catal. - B Enzym.* **5**, 403–409 (1998).
12. Sielaff, B. & Andreesen, J. R. Kinetic and binding studies with purified

recombinant proteins ferredoxin reductase, ferredoxin and cytochrome P450 comprising the morpholine mono-oxygenase from *Mycobacterium* sp. strain HE5. *FEBS J.* **272**, 1148–1159 (2005).

13. Knorr, S. *et al.* Widespread bacterial lysine degradation proceeding via glutarate and L-2-hydroxyglutarate. *Nat. Commun.* **9**, 1–10 (2018).
14. Mande, S. S., Sarfaty, S., Allen, M. D., Perham, R. N. & Hol, W. G. J. Protein–protein interactions in the pyruvate dehydrogenase multienzyme complex: dihydrolipoamide dehydrogenase complexed with the binding domain of dihydrolipoamide acetyltransferase. *Structure* **4**, 277–286 (1996).
15. Campanaro, S. *et al.* New insights from the biogas microbiome by comprehensive genome-resolved metagenomics of nearly 1600 species originating from multiple anaerobic digesters. *Biotechnol. Biofuels 2020 131* **13**, 1–18 (2020).
16. Sery, A. *et al.* Crystal structure of the ferredoxin I from *Desulfovibrio africanus* at 2.3 A resolution. *Biochemistry* **33**, 15408–15417 (1994).
17. Iwaki, H., Hasegawa, Y., Wang, S., Kayser, M. M. & Lau, P. C. K. Cloning and characterization of a gene cluster involved in cyclopentanol metabolism in *Comamonas* sp. strain NCIMB 9872 and biotransformations effected by *Escherichia coli*-expressed cyclopentanone 1,2-monooxygenase. *Appl. Environ. Microbiol.* **68**, 5671–5684 (2002).
18. Chen, Y. C., Peoples, O. P. & Walsh, C. T. *Acinetobacter cyclohexanone* monooxygenase: gene cloning and sequence determination. *J. Bacteriol.* **170**, 781–789 (1988).
19. Iwaki, H. *et al.* Identification of a transcriptional activator (ChnR) and a 6-oxohexanoate dehydrogenase (ChnE) in the cyclohexanol catabolic pathway in *Acinetobacter* sp. strain NCIMB 9871 and localization of the genes that encode them. *Appl. Environ. Microbiol.* **65**, 5158–5162 (1999).
20. Choi, J. H. *et al.* Cloning and characterization of cyclohexanol dehydrogenase gene from *Rhodococcus* sp. TK6. *J. Microbiol. Biotechnol.* **15**, 1189–1196 (2005).
21. Takehara, I. *et al.* Metabolic pathway of 6-aminohexanoate in the nylon oligomer-degrading bacterium *Arthrobacter* sp. KI72: identification of the enzymes responsible for the conversion of 6-aminohexanoate to adipate. *Appl. Microbiol. Biotechnol.* **102**, 801–804 (2018).

22. Yamanishi, Y. *et al.* Prediction of missing enzyme genes in a bacterial metabolic network: Reconstruction of the lysine-degradation pathway of *Pseudomonas aeruginosa*. *FEBS J.* **274**, 2262–2273 (2007).
23. Steinmetz, C. G., Xie, P., Weiner, H. & Hurley, T. D. Structure of mitochondrial aldehyde dehydrogenase: the genetic component of ethanol aversion. *Structure* **5**, 701–711 (1997).
24. Brzostowicz, P. C., Walters, D. M., Thomas, S. M., Nagarajan, V. & Rouvière, P. E. mRNA differential display in a microbial enrichment culture: Simultaneous identification of three cyclohexanone monooxygenases from three species. *Appl. Environ. Microbiol.* **69**, 334–342 (2003).
25. Duarte, M. *et al. Gulosibacter molinativorax* ON4^T molinate hydrolase, a novel cobalt-dependent amidohydrolase. *J. Bacteriol.* **193**, 5810–5816 (2011).
26. Pacheco, C. C. *et al.* Modulation of intracellular O_2 concentration in *Escherichia coli* strains using oxygen consuming devices. *ACS Synth. Biol.* **7**, 1742–1752 (2018).
27. Gabdulkhakov, A. *et al.* Investigations of accessibility of T2/T3 copper center of two-domain laccase from *Streptomyces griseoflavus* ac-993. *Int. J. Mol. Sci.* **20**, (2019).
28. Stackebrandt, E., Stackebrandt, E., Goodfellow, M., Goodfellow, M. & Stackebrandt, E. *Nucleic acid techniques in bacterial systematics*. *Nucleic acid techniques in bacterial systematics* (Wiley, 1991).
29. Delong, E. F., Taylor, L. T., Marsh, T. L. & Preston, C. M. Visualization and enumeration of marine panktonic Archaea and Bacteria by using polyribonucleotide probes and fluorescent *in situ* hybridization. *Appl. Environ. Microbiol.* **65**, 5554–5563 (1999).
30. Hunt, D. E. *et al.* Evaluation of 23S rRNA PCR primers for use in phylogenetic studies of bacterial diversity. *Appl. Environ. Microbiol.* **72**, 2221–2225 (2006).
31. Hunter, S. B. *et al.* Establishment of a universal size standard strain for use with the pulsenet standardized pulsed-field gel electrophoresis protocols: Converting the national databases to the new size standard. *J. Clin. Microbiol.* **43**, 1045–1050 (2005).
32. Sullivan, M. J., Petty, N. K. & Beatson, S. A. Easyfig: A genome comparison visualizer. *Bioinformatics* **27**, 1009–1010 (2011).
